# Actor Power and Perceived Partner Power Differentially Relate to Sexual Behavior and Motivations

**DOI:** 10.1177/01461672251390006

**Published:** 2025-11-27

**Authors:** Nickola C. Overall, Jessica A. Maxwell, Amy Muise, Nina Waddell, Auguste G. Harrington

**Affiliations:** 1 The University of Auckland, New Zealand; 2 McMaster University, Hamilton, ON, Canada; 3 York University, Toronto, ON, Canada; 4 New York University Abu Dhabi, United Arab Emirates

**Keywords:** power, sexual assertiveness, sexual compliance, accommodation, communal motivation

## Abstract

Assertively pursuing sexual needs versus complying or accommodating to partner’s sexual desires has considerable implications. We apply and integrate general theories of power to identify how people’s own power (actor power) and perceptions of their partner’s power (perceived partner power) differentially relate to sexual behaviors and motivations in woman–man relationships. Across three studies (total *N* = 995), actors’ power predicted sexual approach–inhibition: actors higher in power reported more comfort initiating and refusing sex, more assertive sexual communication, and less sexual compliance. By contrast, perceived partner power predicted sexual accommodation–neglect: when partners were perceived to be higher in power, actors expressed greater willingness to compromise and sexual communal strength, and greater understanding when partners rejected sex. These distinct effects were not magnified by asymmetries in perceived power, nor did they differ across gender. Facilitating actor and partner power in intimate relationships should reduce rather than amplify harmful sexual behavior.

Relationship power—the perceived capacity to influence partners—determines whether people can freely pursue their needs or feel compelled to prioritize their partner’s needs (Anderson et al., 2012; Keltner et al., 2003). Power has considerable implications within the sexual domain. Believing one has power enables actors to confidently pursue their sexual needs (Hanna-Walker et al., 2024; Lammers & Stoker, 2019) but such assertive pursuit could risk neglecting their partner’s desires (Kunstman & Maner, 2011). Believing one lacks power, in contrast, may lead actors to inhibit their needs and comply to undesired sexual activity (Conroy et al., 2015; Himanen & Gunst, 2024). Sexual assertiveness is fundamental to healthy, satisfying sexual relationships (Couture et al., 2025; Zhang et al., 2022) but neglecting or complying with partner’s desires can have harmful repercussions (Katz & Tirone, 2009; Nickull et al., 2025).

Despite crucial implications, how power relates to these important sexual behaviors is unclear. Prior studies have examined single behaviors in isolation, such as sexual assertiveness or compliance, and overlooked other key power-relevant behaviors, such as accommodating or neglecting partners’ sexual needs. In addition, assessments of power vary across studies and obscure whose power is motivating different behaviors. Sexual compliance may arise from actors lacking power to assert their needs (Himanen & Gunst, 2024), or being motivated to meet the needs of partners perceived high in power (Conroy et al., 2015; Katz & Tirone, 2009), or both. Similarly, neglecting partners’ sexual desires might arise from high power actors pursuing their own needs (Lammers & Stoker, 2019), or because actors are less motivated to meet the needs of partners perceived low in power (Livingston & Vik, 2021), or both. Finally, because the presence and implications of power and sexual behavior may be gendered, many studies have only focused on women (e.g., Conroy et al., 2015; Himanen & Gunst, 2024).

The current studies address these limitations to provide the most comprehensive examination of power and sexual behavior and motivations to date. We apply a new model (Overall et al., 2023; Overall & Hammond, 2026) to clarify how actor and perceived partner power should relate to distinct sexual behaviors. We then present three studies that test how actor power and perceived partner power relate to a range of sexual behaviors and motivations for both women and men involved in woman–man relationships (see Table 1). Across studies we test whether the effects of power on sexual behaviors and motivations depend on asymmetries between actor and perceived partner power and/or are modified by gender.

**Table 1.** Predicted Associations Between Actor Power and Perceived Partner Power and Sexual Behaviors and Motivations.

Source of power	Psychological process	Sexual behaviors and motivations
Actor Power	• Actors high in power feel able to influence partners to achieve goals and negotiate undesired outcomes, allowing actors to confidently approach and pursue their needs and desires • Actors low in power feel unable to influence partners to negotiate undesired outcomes, prompting actors to inhibit their needs and desires	Higher versus lower actor power will be associated with higher sexual approach versus inhibition • Higher versus Lower Comfort Initiating and Refusing Sex (Studies 1 and 3) • Higher versus Lower Sexual Assertiveness (Studies 2 and 3) • Lower versus Higher Sexual Compliance (Studies 2 and 3)
Perceived Partner Power	• Partners high in power are perceived to influence actors’ ability to attain their needs and goals, prompting actors to accommodate their partners’ needs and preferences • Partners low in power are perceived to have less influence on actors’ desired outcomes, allowing actors to neglect their partner’s needs and desires	Higher versus lower perceived partner power will be associated with higher sexual accommodation versus neglect • Higher versus Lower Enacted Compromise* and Willingness to Compromise (Studies 1–3) • Higher versus Lower Sexual Communal Strength (Studies 2 and 3) • Higher versus Lower Sexual Understanding (Study 3) • Lower versus Higher Sexual Enticement* (Study 3)

*Note.* The results across studies supported these distinctions except for the two variables marked *. Lower actor power rather than higher perceived partner power predicted greater enacted compromise in two of three studies (see Discussion about the relevance of the distinction between willingness and enacted compromise). In addition, perceived partner power (nor actor power or the interaction between actor and partner power) was not significantly associated with sexual enticement, a more extreme form of partner neglect involving directly ignoring partners’ needs to pursue actors’ own desires.

## Actor and Perceived Partner Power: Definitions and Distinct Behaviors and Motivations

Many factors—personal characteristics, relationship commitment, resources, expertise—shape people’s power in relationships (Simpson et al., 2015). However, these sources of power shape behavior via actors’ perceived capacity to influence their partners (e.g., “I can get my partner to do what I want,” “I can get my partner to listen to what I say”; Anderson et al., 2012). For example, relational (e.g., commitment, attachment) and resource (e.g., SES, education, income) bases of power each contribute to actors’ perceptions of their power, but perceived power most strongly predicts relationship outcomes (e.g., Körner & Schütz, 2021; Overall et al., 2023). Thus, as emphasized by central theories, power-relevant behaviors emerge from actors’ perceived capacity to influence rather than whether actors can or do enact influence in any given situation (Anderson et al., 2012; Galinsky et al., 2015; Simpson et al., 2015). Actors who report higher power believe they can negotiate outcomes if needed and thus confidently approach their desires often without needing to exert influence, whereas actors who report low power believe they do not have the influence to negotiate any negative consequences and so inhibit their desires often without trying to influence (Keltner et al., 2003; Overall et al., 2023). Finally, power can vary across specific domains (e.g., finances, relationship issues, sex), but reports of general relationship power highly correlate with domain-specific power and as strongly predict behavior within specific domains (Farrell et al., 2015). Thus, our theoretical and empirical analyses focus on actors’ reports of their general power to influence partners to achieve desired outcomes (Anderson et al., 2012).

Prior studies examining how relationship power shapes sexual behavior have similarly assessed reported power, although most measures have confounded actor power with perceived partner power by operationalizing actor power as perceptions of partners’ decision-making power or controlling behavior (e.g., Conroy et al., 2015; Himanen & Gunst, 2024), or equal versus unequal perceived power or decision-making across actors and partners (e.g., Brezsnyak & Whisman, 2004; Forbush et al., 2025; Hanna-Walker et al., 2024). These assessments obfuscate the effects of power because, although often conceptualized in relative terms (i.e., actors have less power when partners have more power), growing evidence shows that actor and partner power are distinct and have differential effects (Overall & Hammond, 2026). For example, studies separately assessing actor power and partner power (perceived by actors or reported by partners) reveal these are modestly *positively* correlated rather than inversely related as is assumed when measuring low actor power by assessing partner’s higher power (e.g., Farrell et al., 2015; Körner & Schütz, 2024; Overall et al., 2023). The associations between actor and partner power are modest, however, because relationships can involve actors and partners both having high power (mutually influencing each other), both having low power (lacking influence over each other), or one having more power than the other (e.g., Hanna-Walker et al., 2024).

Critically, regardless of whether levels of power are similar or different, studies separately assessing actor and partner power have shown actor and partner power have distinct behavioral consequences (e.g., Gresham et al., 2023; Körner et al., 2022; Overall et al., 2023). Overall et al. (2023) integrated the approach-inhibition theory (Keltner et al., 2003) and agentic-communal model (Rucker & Galinsky, 2016) of power to develop a framework for understanding these distinct patterns (also see Overall & Hammond, 2026). Actors’ own power determines approach–inhibition: actors who report high power confidently pursue their needs and goals (approach), whereas low power actors inhibit their needs and goals (inhibition). Partners’ power determines accommodation–neglect: actors show greater care, compromise, and support (accommodation) for partners perceived high in power, but less care and support (neglect) for partners perceived low in power (see Overall & Hammond, 2026). We apply this framework to specify how actor and perceived partner power should relate to different sexual behaviors and motivations (see Table 1).

### Actor Power and Sexual Approach–Inhibition

Actors’ own power determines whether they confidently approach or inhibit their needs and goals (Overall et al., 2023; Overall & Hammond, 2026). Actors high in power feel able to influence their partner to achieve desired goals and negotiate undesired outcomes, promoting confident pursuit of their needs (Galinsky et al., 2015). Accordingly, actors who report high power feel more capable of managing challenging interactions (Gresham et al., 2023), feel less threatened when partners are rejecting or hostile (Kuehn et al., 2015), and use direct constructive strategies for managing conflict (Farrell et al., 2015). By contrast, actors low in power believe they lack the influence needed to manage undesirable consequences, prompting inhibition of behavior that may risk negative outcomes (Keltner et al., 2003). Accordingly, actors who report low power inhibit the expression of negative thoughts and feelings, especially during threatening interactions (Alonso-Ferres et al., 2021; Overall et al., 2023; Pietromonaco et al., 2021).

Approach versus inhibition is important in vulnerable contexts where people must express their desires to fulfill their needs, but doing so could incite rejection or hostility. Initiating sex or communicating sexual desires risks partners’ rebuff, and declining partners’ sexual advances or preferred sexual activities risks partners’ dissatisfaction and hostility (Impett et al., 2020). High power actors, however, believe they have the influence needed to protect themselves and encourage partner responsiveness in embarrassing or dissatisfying situations. Thus, actors who report higher power should feel more confident approaching their partner to initiate sex and more comfortable expressing their needs including rejecting sex when undesired. Actors who report low power, in contrast, believe they lack influence to manage rejection or embarrassment and thus should be more inhibited, including feeling less comfortable initiating sex and expressing sexual desires. Moreover, because they believe they lack influence to negotiate partner’s dissatisfaction, low power actors should feel less comfortable refusing sex and be more likely to consent to or engage in undesired sexual activity—sexual compliance (Impett & Peplau, 2003).

Some evidence supports that high versus low actor power will promote sexual approach versus inhibition. Greater reported power at work predicts greater sexual assertiveness in relationships (Lammers & Stoker, 2019), and women’s higher reported relationship power is associated with greater sexual assertiveness (Hanna-Walker et al., 2024). By contrast, women who are concerned that they lack power in sexual encounters report less sexual assertiveness (Zerubavel & Messman-Moore, 2013), women who report lower decision-making power relative to their partner report greater compliance to undesired sexual activity (Conroy et al., 2015; Himanen & Gunst, 2024), and perceived power struggles are associated with inhibited sexual passion (Forbush et al., 2025). These studies, however, have (a) not measured assertiveness and compliance in the same studies, (b) used various power measures that obscure whether assertiveness or compliance arise from actor power rather than—or in conjunction with—perceived partner power, and (c) primarily shown or only assessed effects for women. As summarized in Table 1, in three studies we test whether high versus low actor power is distinctly associated with women’s and men’s sexual approach–inhibition, including comfort initiating and refusing sex, sexual assertiveness, and sexual compliance.

### Perceived Partner Power and Sexual Accommodation–Neglect

Perceived partner power relates to whether actors accommodate or neglect their partner’s needs and goals (Overall et al., 2023; Overall & Hammond, 2026). Partners have more power to exert influence when actors depend on partners for valued outcomes (Kelley & Thibaut, 1978) compelling actors to be more accommodating of high power partners (also Rucker & Galinsky, 2016). For example, perceiving partners to hold higher power is associated with sacrificing more for partners (Körner et al., 2022; Righetti et al., 2015) and adopting partners’ goals (Laurin et al., 2016). Conversely, actors are less concerned about accommodating partners they perceive low in power as they feel able to attain their goals without their partner’s cooperation. Thus, when faced with conflicting interests, actors are more likely to neglect the needs of partners perceived low in power, expressing less care, support, and compromise (Overall et al., 2023; Righetti et al., 2015).

Accommodating partners’ needs and compromising when diverging desires arise helps maintain satisfying relationships (Righetti et al., 2020), whereas neglecting partners’ needs (less care, support, compromise) harms partners’ satisfaction and wellbeing (Le et al., 2018). Sexual accommodation versus neglect is equally consequential. Actors motivated to be responsive to their partner’s sexual needs—called sexual communal strength—are more accommodating of their partners’ needs during sex (Muise & Impett, 2015), more understanding when their partner does not want sex (Muise, Kim, et al., 2017), and more effectively manage sexual difficulties or disagreements in ways that sustain actors’ and partners’ relationship quality (Day et al., 2015; Muise, Bergeron, et al., 2017). By contrast, greater neglect of partner’s sexual needs—lower sexual communal strength—undermines couples’ sexual and relationship satisfaction (also see Balzarini et al., 2021).

Some suggestive evidence supports that perceiving high versus low partner power will promote sexual accommodation versus neglect. Showing accommodation of partners’ sexual desires, for example, couples tend to report contraceptive practices that align with the preferences of high power partners (VanderDrift et al., 2013), likely accounting for why young women report less consistent condom use when they perceive partners as higher in power (e.g., Kogan et al., 2013). No prior studies, however, have directly examined whether perceived partner power motivates actors to be more accommodating of their partner’s sexual preferences beyond contraceptive use, such as compromising with their partner about how often or how they engage in sex. Prior studies also have not assessed the underlying motivation to meet the partner’s needs or whether perceiving low partner power promotes neglect of partners’ needs or desires, such as continuing to entice partners rather than expressing understanding when partners express they do not want sex. In the current studies, we test whether perceived partner power is distinctly associated with sexual accommodation–neglect, including sexual compromise with partners, sexual communal strength, and sexual understanding versus enticement when partners reject sex (see Table 1).

## The Possible Role of Asymmetries in Perceived Power and Gender

Many theories and empirical investigations of power have focused on asymmetries implying that power shapes behavior primarily when actors experience higher power than partners, or vice versa (Galinsky et al., 2015). Yet, as described above, actor and partner power are related to distinct psychological processes that do not hinge on power asymmetries. For example, high power actors should feel able to express their needs and initiate sex regardless of whether they perceive (a) their partners high in power and thus actors are motivated to also accommodate their partner’s needs, or (b) their partner is low in power and actors may neglect their partner’s needs. As in other recent studies demonstrating distinct effects of actor and partner power (e.g., Gresham et al., 2023; Körner & Schütz, 2024; Körner et al., 2022; Overall et al., 2023), we expected that actor power would predict sexual approach–inhibition and perceived partner power would predict sexual accommodation–neglect regardless of whether actor and perceived partner power were both low or high, or were discrepant, in levels of power.

It remains possible, however, that the combination of the psychological processes associated with actor and perceived partner power amplify the potential for harmful sexual behaviors. For example, the effect of low actor power on inhibition (lower assertiveness in initiating sex and communicating desires) may be magnified when high perceived partner power motivates accommodation of partners’ needs (greater compromise and communal strength), producing greater risk of sexual compliance. Indeed, studies examining women’s motivations (Conroy et al., 2015; Impett & Peplau, 2003; Katz & Tirone, 2009) suggest that sexual compliance can involve wanting to avoid conflict and partners’ discontent (inhibition) and promote partners’ pleasure and intimacy (accommodation). Thus, to test the role of power asymmetries, we include interactions between actor and perceived partner power in all analyses.

Finally, biological and socialization differences may generate gender differences. For example, men’s greater sexual drive (Frankenbach et al., 2022) combined with sexual scripts that stipulate men as dominant sexual agents (Wiederman, 2015) suggest men will be more sexually assertive (Hanna-Walker et al., 2024; Impett & Peplau, 2003). By contrast, gendered sexual scripts and traditional gender roles stipulate that women should be sexually submissive and nurturing caregivers which may lead women to be more inhibited, compliant, and accommodating of partners’ sexual desires (Conroy et al., 2015; Impett & Peplau, 2003; Zerubavel & Messman-Moore, 2013). However, many studies examining power and sexual behavior have only included women, assuming rather than demonstrating gender differences. By contrast, we assess both women’s and men’s power and sexual behavior and test for gender differences across analyses.

## Research Overview

We conducted three studies to test the predicted associations between actor and perceived partner power and sexual behaviors and motivations in Table 1. In each study, participants reported their own power (actor power), perceptions of their partner’s power (perceived partner power), and behaviors and motivations that reflect sexual approach–inhibition or accommodation–neglect (see Table 1). Study 1 provided an initial test using an existing dataset that included measures of sexual approach–inhibition (comfort initiating and refusing sex) and accommodation–neglect (sexual compromise). Study 2 aimed to replicate the predicted associations using a more detailed measure of sexual approach (sexual assertiveness), targeted assessment of inhibition (sexual compliance), and an additional measure of accommodation–neglect (sexual communal strength). Study 3 included all measures in Studies 1 and 2 plus additional measures of sexual compliance (sexual acquiescence), accommodation (sexual understanding), and a more extreme form of neglect (enticement when partners decline sex).

All studies included women and men in woman–man relationships because this relationship context specifically relates to the sexual scripts and gender roles that may produce gender differences in how power shapes sexual behavior. Our primary analyses modeled actor’s own reported power and perceptions of their partner’s power as simultaneous predictors to test whether actor power predicted sexual approach–inhibition and perceived partner power predicted accommodation–neglect. We also included interactions between actor and perceived partner power and the main and interaction effects of gender to assess whether the links between power and sexual behavior and motivations depended on power asymmetries or differed across women and men. Finally, because higher power may predict greater sexual satisfaction and desire (Brezsnyak & Whisman, 2004; Körner & Schütz, 2024), and sexual behavior and motivations shape sexual satisfaction and desire (Impett et al., 2020), we ran additional analyses to ensure the effects of power were independent of sexual satisfaction and desire.

## Transparency and Openness

Study 1 was not pre-registered. Studies 2 and 3 were pre-registered. Pre-registrations and data, syntax, and measures for all studies are available at https://osf.io/e54zr/. We report all exclusions. The University of Auckland Human Participants Ethics Committee approved each study.

## Study 1

Participants completed measures of their own and their partner’s perceived power, comfort initiating and refusing sex, and sexual compromise (see Table 1).

### Method

#### Participants and Procedure

Data were drawn from an online study on Prolific.com which compensated £4.17 for a 40-min questionnaire and required participants to be sexually active, in committed man–woman relationships, childfree, and over 18. Participants were included if they identified as a heterosexual man or woman. We excluded 22 participants who failed attention checks. The sample of 270 participants (130 women, 140 men) provides 80% power to detect small effects (*f*^2^ = .029; β = .17). Supplemental Material (SM) provides additional information.

Table 2 presents demographics. Participants were in well-established relationships (>60% cohabiting or married). The majority resided in the United Kingdom and identified as White/Caucasian.

**Table 2.** Sample Demographics.

	*Study 1* (N = 270)		*Study 2* (N = 304)		*Study 3* (N = 412)	
Variable	*M*	*SD*	*M*	*SD*	*M*	*SD*
Age (years)	30.77	9.69	28.03	6.38	33.78	10.15
Relationship length (years)	6.89	6.79	4.98	3.92	7.79	6.80
*Gender*	*n*	*%*	*N*	*%*	*n*	*%*
Women	130	48	152	50	211	51
Men	140	52	152	50	210	49
*Relationship status*	*n*	*%*	*n*	*%*	*n*	*%*
Casual/dating					2	.5
Steady/exclusive dating	13	4.8	2	.07	31	7.4
Serious	83	30.7	23	7.60	91	21.6
Living together	104	38.5	177	58.2	154	36.6
Married	67	24.8	88	28.9	131	31.1
Other (e.g., Engaged)	3	1.1	14	4.6	12	2.9
*Country of residence*	*n*	*%*	*N*	*%*	*n*	*%*
United Kingdom and Northern Ireland	171	63.3	-	-	197	46.8
United States of America	60	22.2	-	-	167	39.7
Canada	25	9.3	-	-	57	13.5
Australia or New Zealand	7	2.6	304	100	-	-
Other European	7	2.7	-	-	-	-
*Ethnic group identificationa*	*n*	*%*	*n*	*%*	*n*	*%*
*Studies 1 and 3*						
White/Caucasian	231	85.6	-	-	303	72.0
East Asian	14	5.2	-	-	27	6.4
South Asian	8	3.0	-	-	23	5.5
South East Asian	5	1.9	-	-	15	3.6
Black/African-American	8	3.0	-	-	23	5.5
Latin American/Hispanic	5	1.9	-	-	16	3.8
African	2	0.7	-	-	17	4.0
Indigenous/Aboriginal	2	0.7	-	-	1	0.2
Arab/West Asian	-	-	-	-	5	1.2
Study 2						
NZ European/Pakeha	-	-	156	51.3	-	-
Non-NZ European	-	-	57	18.8	-	-
Māori	-	-	21	6.9	-	-
Pacific Nations	-	-	5	1.6	-	-
Asian	-	-	55	18.1	-	-
Indian	-	-	24	7.9	-	-
Another ethnicity	8	3.0	38	12.5	10	2.4

*Note.* “-” denotes locations or ethnic group not sampled in study. ^a^Ethnic groups varied across location of the study; participants in each study could select multiple ethnic groups.

#### Measures

##### Actor Power

Participants completed the eight-item relationship-specific Sense of Power Scale (Anderson et al., 2012; for example, In my relationship . . . “I think I have a great deal of power,” “Even when I try, I am not able to get my way” [reverse coded]; 1= *strongly disagree*, 7 = *strongly agree*). Items were averaged (α = .86).

##### Perceived Partner Power

As in other studies (e.g., Körner & Schütz, 2024; Overall et al., 2023), participants also completed the Sense of Power Scale worded to assess perceptions of partners’ power (e.g., “I think my partner has a great deal of power”; α = .80).

##### Sexual Approach–Inhibition

Participants rated their comfort in (a) initiating sex and (b) refusing sex in their current relationship using two face-valid items: “I am comfortable initiating sex” and “I am comfortable refusing sex” (1 = *strongly disagree*, 7 = *strongly agree*).

##### Sexual Accommodation–Neglect

Participants completed scales assessing sexual compromise (adapted from Burke & Young, 2012). We focused on items regarding frequency and types of sexual activities because these compromises are often relevant to man–woman couples (MacNeil & Byers, 1997; see SM for further details). Participants rated their *enacted compromise* by indicating how frequently they had compromised with their partner regarding “frequency of sexual activity,” and “types of sexual activities engaged in (e.g., oral sex, sexting)” over the past 6 months (1 = *never compromised*, 7 = *regularly compromised*). Participants then rated their *willingness to compromise* for both frequency and type of sexual activity (1= *not at all willing*, 7= *extremely* willing). Participants could select “not applicable” for each item reducing the sample for analyses by 17 for enacted and 4 for willingness. We averaged items to reduce the number of analyses (enacted *r* = .36; willingness *r* = .58); analyzing each item produced the same results.

##### Additional Analyses Measure

Participants completed a five-item sexual satisfaction measure (Lawrance & Byers, 1995; see SM).

### Results

Table 3 presents descriptives (SM presents correlations). To test the distinct effects of actor and perceived partner power and the possibility of asymmetries in perceived power and gender differences, we conducted multiple regression analyses using SPSS version 29. We regressed each outcome on: actor power, perceived partner power, the interaction between actor power and perceived partner power, and all main and interaction effects of gender.

**Table 3.** Descriptive Information for Primary Measures.

Variable	*Scale range*	*Study 1*		*Study 2*		*Study 3*	
		*Mean (SD)*	*Range*	*Mean (SD)*	*Range*	*Mean (SD)*	*Range*
Actor Power	1-7	5.24 (0.97)	2.13-7.00	5.38 (1.00)	2.25-7.00	5.31 (0.97)	1.75-7.00
Perceived Partner Power	1-7	5.59 (0.76)	3.25-7.00	5.79 (0.82)	2.50-7.00	5.65 (0.84)	3.00-7.00
*Sexual Approach–Inhibition*							
Comfort Initiating Sex	1-7	5.73 (1.37)	1.00-7.00	-	-	5.67 (1.58)	1.00-7.00
Comfort Refusing Sex	1-7	5.43 (1.47)	1.00-7.00	-	-	5.32 (1.71)	1.00-7.00
Sexual Assertiveness	0-4	-	-	2.85 (0.65)	1.13-4.00	2.87 (0.73)	0.56-4.00
Sexual Compliance	0-4	-	-	0.94 (0.74)	0.00-3.67	0.81 (0.76)	0.00-3.67
Sexual Acquiescence	0-4	-	-	-	-	0.36 (0.60)	0.00-3.20
*Sexual Accommodation–Neglect*							
Enacted Compromise	1-7	3.81 (1.72)	1.00-7.00	3.76 (1.68)	1.00-7.00	3.72 (1.84)	1.00-7.00
Willingness to Compromise	1-7	5.67 (1.35)	1.00-7.00	5.60 (1.25)	1.00-7.00	5.77 (1.26)	1.00-7.00
Communal Strength	1-7	-	-	5.64 (0.87)	2.50-7.00	5.72 (0.96)	2.25-7.00
Sexual Understanding	1-5	-	-	-	-	3.83 (0.98)	1.00-5.00
Sexual Enticement	1-5	-	-	-	-	1.98 (0.92)	1.00-5.00

*Note.* “-” denotes measured not assessed in study.

#### Sexual Approach–Inhibition

As shown in Table 4, higher versus lower actor power was associated with greater versus lower comfort in initiating and refusing sex, whereas perceived partner power was not associated with approach–inhibition. The Actor × Perceived Partner Power interaction was significant when predicting comfort refusing sex. Decomposing the interaction revealed that the positive link between actor power and comfort refusing sex was stronger when perceived partner power was high (+1 *SD: b* = .60, *t* = 5.48, *p* < .001) versus low (−1 *SD: b* = .29, *t* = 2.02, *p* = .045). Rather than power asymmetries playing a role, actors were most comfortable refusing sex when both actor power and perceived partner power were high (see SM for further details). None of the main or interaction effects of power differed across gender.

**Table 4.** Associations Between Actor Power, Perceived Partner Power, and Comfort Initiating and Refusing Sex (Studies 1 and 3).

Predictors	*Comfort initiating sex*					*Comfort refusing sex*				
	*b*	95% CI	β	*t*	*p*	*b*	95% CI	β	*t*	*p*
*Study 1*										
Actor Power	**.35**	**.16, .53**	**.25**	**3.67**	**<.001**	**.45**	**.25, .64**	**.30**	**4.49**	**<.001**
Perceived Partner Power	.23	-.01, .46	.13	1.91	.057	.05	-.20. .29	.02	0.38	.703
Actor Power × Perceived Partner Power	-.01	-.21, .18	-.01	-0.14	.886	**.21**	**.00, .41**	**.12**	**1.97**	**.050**
Gender	.11	-.06, .28	.08	1.33	.186	-.18	-.36, -.01	-.13	-2.04	.042
Gender × Actor Power	.12	-.07, .31	.08	1.28	.202	-.16	-.36, .03	-.10	-1.62	.106
Gender × Perceived Partner Power	.01	-.22, .24	.01	0.10	.921	.10	-.14, .34	.05	0.81	.343
Gender × Actor Power × Perceived Partner Power	-.15	-.35, .05	-.10	-1.53	.128	-.07	-.28, .14	-.04	-0.65	.516
*Study 3*										
Actor Power	**.35**	**.18, .53**	**.22**	**3.96**	**<.001**	**.29**	**.11, .48**	**.17**	**3.07**	**.002**
Perceived Partner Power	.03	-.18, .23	.02	0.27	.785	.18	-.04. .40	.09	1.62	.105
Actor Power × Perceived Partner Power	.04	-.13, .22	.02	0.50	.617	**.24**	**.06, .43**	**.12**	**2.57**	**.011**
Gender	.31	.15, .48	.20	3.81	<.001	-.45	-.62, -.28	-.26	-5.13	<.001
Gender × Actor Power	.03	-.15, .21	.02	0.34	.731	.02	-.16, .21	.01	0.25	.807
Gender × Perceived Partner Power	.03	-.18, .24	.02	0.29	.772	.05	-.17, .26	.02	0.41	.683
Gender × Actor Power × Perceived Partner Power	.03	-.14, .21	.02	0.37	.716	**.21**	**.02, .39**	**.12**	**2.20**	**.028**

*Note*. Significant effects involving power are shown in bold. Gender = −1 women, 1 men.

#### Sexual Accommodation–Neglect

As shown in Table 5, greater versus lower perceived partner power was associated with greater versus lower enacted and willingness to compromise, whereas actors’ own power did not have significant independent effects. The Actor × Perceived Partner Power interaction was significant for willingness to compromise. The effect of perceived partner power on greater willingness to compromise was stronger when actor power was high (+1 *SD: b* = .77, *t* = 4.74, *p* < .001) versus low (−1 *SD: b* = .31, *t* = 2.36, *p* = .019). Thus, rather than evidencing power asymmetries, compromise was greatest when both perceived partner power and actor power were high (SM provides further details). None of the main or interaction effects significantly differed across gender.

**Table 5.** Associations Between Actor Power, Perceived Partner Power, and Sexual Compromise (Studies 1–3).

Predictors	*Enacted compromise*					*Willingness to compromise*				
	*B*	95% CI	β*/r*	*t*	*p*	*b*	95% CI	β*/r*	*t*	*p*
*Study 1*										
Actor Power	-.07	-.33, .18	-.04	-0.58	.564	.08	-.10, .26	.06	0.90	.367
Perceived Partner Power	**.48**	**.17, .79**	**.21**	**3.02**	**.003**	**.54**	**.32, .76**	**.31**	**4.74**	**<.001**
Actor Power × Perceived Partner Power	.16	-.10, .42	.08	1.22	.223	**.24**	**.05, .43**	**.15**	**2.44**	**.015**
Gender	.17	-.06, .40	.10	1.48	.141	.32	.16, .48	.24	3.85	<.001
Gender × Actor Power	-.20	-.45, .05	-.11	-1.59	.113	-.09	-.27, .09	-.06	-0.99	.325
Gender × Perceived Partner Power	-.04	-.35, .27	-.02	-0.26	.769	-.08	-.30, .15	-.04	-0.68	.495
Gender × Actor Power × Perceived Partner Power	.02	-.24, .28	.01	0.16	.876	-.05	-.24, .14	-.04	-0.54	.587
*Study 2*										
Actor Power	**-.32**	**-.53, -.11**	**-.18**	**-3.00**	**.003**	.05	-.10, .21	.04	0.69	.489
Perceived Partner Power	-.07	-.33, .18	-.03	-0.58	.566	**.24**	**.05, .42**	**.15**	**2.54**	**.012**
Actor Power × Perceived Partner Power	.04	-.18, .27	.02	0.39	.695	.04	-.13, .20	.03	0.43	.670
Gender	.13	-.07, .33	.10	1.28	.204	.15	.01, .29	.16	2.06	.041
Gender × Actor Power	-.04	-.25, .17	-.03	-0.42	.678	-.06	-.21, .09	-.05	-0.79	.428
Gender × Partner Perceived Power	.03	-.23, .28	.01	0.20	.844	.13	-.05, .31	.08	1.38	.167
Gender × Actor Power × Perceived Partner Power	-.03	-.25, .19	-.02	-0.29	.775	.05	-.12, .21	.03	0.54	.590
*Study 3*										
Actor Power	**-.23**	**-.45, -.01**	**-.12**	**-2.10**	**.037**	.07	-.07, .21	.05	0.98	.326
Perceived Partner Power	.15	-.10, .41	.07	1.17	.243	**.32**	**.16, .48**	**.21**	**3.91**	**<.001**
Actor Power × Perceived Partner Power	.03	-.18, .25	.01	0.28	.782	.00	-.14, .14	.00	-0.01	.997
Gender	.10	-.10, .30	.06	1.00	.316	.28	.16, .41	.22	4.36	<.001
Gender × Actor Power	.16	-.06, .38	.08	1.46	.145	**.15**	**.01, .29**	**.11**	**2.12**	**.035**
Gender × Perceived Partner Power	-.24	-.49, .02	-.11	-1.82	.070	-.13	-.29, .03	-.09	-1.59	.113
Gender × Actor Power × Perceived Partner Power	-.13	-.35, .09	-.07	-1.15	.251	**-.16**	**-.30, -.02**	**-.12**	**2.24**	**.025**

*Note*. Significant effects involving power are shown in bold. Gender = −1 women, 1 men. Studies 1 and 3 involved multiple regression analyses of individuals producing β as indicator of standardized effect size. Study 2 involved multilevel regression analyses of dyads with *r* effect sizes calculated as an indicator of effect size.

#### Additional Analyses

Actor and perceived partner power were positively associated with sexual satisfaction, but rerunning the analyses controlling for satisfaction did not alter the significant effects in Tables 3 and 4 with one exception: the link between actor power and comfort initiating sex was no longer significant (see SM).

## Study 2

Study 1 provided initial evidence that actor power predicts sexual approach–inhibition (comfort initiating and refusing sex) whereas perceived partner power predicts sexual accommodation–neglect (enacted and willingness to compromise with partners). Although two (of four) Actor × Perceived Partner Power interactions were significant, these interactions did not provide support for asymmetries in perceived power. Instead, actors’ comfort to refuse sex and willingness to compromise was greatest when both actor power and perceived partner power were high. None of the effects of power differed across gender.

Study 2 aimed to replicate the distinct associations between actor and perceived partner power using more detailed and established measures. For approach–inhibition, we replaced the face-valid items assessing comfort initiating and refusing sex with a longer established measure of sexual assertiveness. We also specifically assessed sexual compliance. Although Study 1 indicated power asymmetries were not critical, we wanted to examine whether asymmetries mattered more when assessing more harmful inhibition behavior. We also included an additional measure of accommodation–neglect: sexual communal strength reflects the motivation to meet a partner’s sexual needs and thus is a key index of sexual accommodation–neglect. We pre-registered our predictions, research question, and measurement decisions.

Finally, Study 2 drew from a couple sample allowing us to examine actors’ perceptions of partners’ power as in Study 1 as well as partners’ own reports of their power. Prior studies reveal that both perceived partner power and partner-reported power are linked to accommodation–neglect (e.g., Körner et al., 2022; Overall et al., 2023). Critically, however, perceptions of partner power more strongly and reliably predict accommodation–neglect because any effects of partner power should occur via perceptions that the partner holds power (Anderson & Berdahl, 2002). For example, accommodation is theorized to emerge to attain valued outcomes and sustain relationships with partners that actors perceive influence those outcomes rather than partners who feel powerful forcing actor’s accommodation (Fiske & Berdahl, 2007; Overall et al., 2023). Thus, our primary analyses focused on perceived partner power as in Study 1 and all prior studies examining relationship power and sexual behavior. And, as pre-registered, we expected the effects of perceived partner power would be stronger than the effects of partner-reported power tested in additional analyses.

### Method

#### Participants and Procedure

Data were drawn from a larger study. Couples residing in New Zealand were compensated NZ$40 for completing questionnaires. Participation required couples to be sexually active, childfree, in relationships of 6 months or longer, and aged 18 or above. As in Study 1, we focused on woman-man couples given our aim to test possible gender differences arising from heterosexual scripts and gender roles in woman–man relationships. The sample of 152 woman-man dyads (*N* = 304) provides ample power (.97) to detect the smallest effect size of actor and perceived partner power in Study 1 (β*/r* = .21). SM provides further information. Table 2 presents demographics. Most couples (87%) were cohabiting or married, and two thirds identified as NZ European/Pakeha or Non-NZ European.

#### Measures

Items were averaged to construct scale scores. Table 3 provides descriptives (SM provides correlations).

##### Actor and Perceived Partner Power

The same scales in Study 1 assessed actor (α = .84) and perceived partner (α = .79) power.

##### Sexual Approach–Inhibition

Participants completed the 25-item Hurlbert Index of Sexual Assertiveness (Hurlbert, 1991). As pre-registered, to assess *sexual assertiveness* we excluded items that did not assess sexual approach toward the partner (e.g., discomfort talking to friends), captured other constructs (insistence, unmitigated communion; see details in SM), or assessed compliance (assessed separately as described next). The 16 items used to index *sexual assertiveness* directly assessed comfort in initiating sex (e.g., “I feel comfortable in initiating sex with my partner,” “I approach my partner for sex when I need it”), and open versus inhibited expression of sexual desires (e.g., “I communicate my sexual desires to my partner,” “It is hard for me to be honest about my sexual feelings” [reverse coded]). Items were rated from 0 = *all of the time* to 4 = *never* and coded so that higher scores represent greater assertiveness (α = .89).

Three items in the Hurlbert Index directly assess engaging in undesired sexual activity: “I find myself having sex when l do not really want it,” “I find myself doing sexual things that I do not like,” “It is hard for me to say no even when l do not want sex.” Items were scored so that higher scores represent greater *sexual compliance* (α = .69). Illustrating these items capture a more extreme form of inhibition than low assertiveness, sexual compliance and assertiveness were modestly correlated (*r* = −.36), aligning with recent studies examining sexual assertiveness removing these items (Grose, 2024) or identifying them as a separate factor assessing unwanted sex (Couture et al., 2025).

##### Sexual Accommodation–Neglect

Participants completed the two items from Study 1 assessing *enacted compromise* (*r* = .42) and *willingness to compromise* (*r* = .49). Participants selecting “not applicable” (17 for enacted, 3 for willingness) slightly reduced the sample for analyses. We used a four-item *sexual communal strength* scale (Balzarini et al., 2021) assessing motivation to meet partners’ sexual needs (e.g., “How far would you be willing to go to meet your partner’s sexual needs?,” “How high a priority for you is meeting the sexual needs of your partner?”; 1 = *not at all*, 7 = *extremely;* α = .70).

##### Additional Analyses Measures

Participants completed scales assessing sexual satisfaction (Shaw & Rogge, 2016) and dyadic sexual desire (Spector et al., 1996). See SM.

### Results

Our analytic approach mimicked Study 1 but applied Kenny et al.’s (2006) guidelines and SPSS syntax for distinguishable dyads to simultaneously calculate the main and interaction effects of actor and perceived partner power accounting for the statistical dependence across dyad members. As in Study 1, we regressed each outcome on: actor power, perceived partner power, the interaction between actor power and perceived partner power, and the main and interaction effects of gender.

#### Sexual Approach–Inhibition

As shown in Table 6, higher versus lower actor power was associated with greater versus lower *sexual assertiveness*, and neither the perceived partner power effect nor Actor × Perceived Partner Power interaction were significant. However, a significant gender difference (see Table 6) revealed the actor effect emerged for men (*b* = .29, 95% CI = [.19, .38], *t* = 5.98, *p* < .001) not women (*b* = .03, 95% CI = [−.08, .14], *t* = 0.57, *p* = .572). As shown in Table 7, higher versus lower actor power predicted lower versus higher *sexual compliance*. There were no significant Perceived Partner Power, Actor × Perceived Partner, or Gender interaction effects.

**Table 6.** Associations Between Actor Power, Perceived Partner Power, and Sexual Assertiveness (Studies 2 and 3).

Predictors	*Sexual assertiveness*				
	*b*	95% CI	*r/*β	*t*	*p*
*Study 2*					
Actor Power	**.16**	**.09, .23**	**.25**	**4.26**	**<.001**
Perceived Partner Power	.06	-.03, .14	.07	1.23	.221
Actor Power × Perceived Partner Power	.01	-.07, .08	.01	0.14	.893
Gender	.06	-.01, .12	.14	1.79	.077
Gender × Actor Power	**.13**	**.06, .20**	**.22**	**3.51**	**<.001**
Gender × Perceived Partner Power	.01	-.08, .09	.01	0.08	.936
Gender × Actor Power × Perceived Partner Power	.03	-.04, .11	.06	0.87	.387
*Study 3*					
Actor Power	**.24**	**.16, .32**	**.32**	**5.98**	**<.001**
Perceived Partner Power	.03	-.07, .12	.03	0.57	.567
Actor Power × Perceived Partner Power	**.11**	**.03, .19**	**.12**	**2.67**	**.008**
Gender	.13	.06, .20	.18	3.48	<.001
Gender × Actor Power	.01	-.07, .09	.01	0.20	.841
Gender × Perceived Partner Power	.04	-.06, .13	.04	0.77	.443
Gender × Actor Power × Perceived Partner Power	-.03	-.11, .05	-.04	-0.76	.447

*Note*. Significant effects involving power are shown in bold. Gender = −1 = women, 1 = men. Study 2 involved dyadic multilevel regression models with *r* calculated as an indicator of effect size. Study 3 involved multiple regression analyses of individuals producing β as indicator of standardized effect size.

**Table 7.** Associations Between Actor Power, Perceived Partner Power, and Sexual Compliance and Sexual Acquiescence (Studies 2 and 3).

Predictors	*Sexual compliance*					*Sexual acquiescence*				
	*b*	95% CI	*r/*β	*t*	*p*	*b*	95% CI	β	*t*	*p*
*Study 2*										
Actor Power	**-.19**	**-.27, -.09**	**-.24**	**-4.20**	**<.001**					
Perceived Partner Power	-.06	-.17, .04	-.07	-1.14	.256					
Actor Power × Perceived Partner Power	-.01	-.10, .09	-.01	-0.84	.933					
Gender	-.09	-.17, -.02	-.18	-2.35	.020					
Gender × Actor Power	-.06	-.15, .03	-.08	-1.40	.164					
Gender × Perceived Partner Power	-.01	-.12, .09	-.02	-0.27	.790					
Gender × Actor Power × Perceived Partner Power	-.05	-.14, .04	-.07	-1.05	.295					
*Study 3*										
Actor Power	**-.11**	**-.19, -.03**	**-.14**	**-2.56**	**.011**	**-.08**	**-.15, -.02**	**-.13**	**-2.44**	**.015**
Perceived Partner Power	**-.15**	**-.25, -.05**	**-.16**	**-2.97**	**.003**	-.05	-.13, .03	-.07	-1.29	.199
Actor Power × Perceived Partner Power	.04	-.04, .13	.05	1.00	.318	.05	-.02, .12	.08	1.54	.125
Gender	-.14	-.22, -.07	-.19	-3.62	<.001	-.07	-.13, -.01	-.11	-2.16	.031
Gender × Actor Power	.05	-.04, .13	.06	1.07	.287	.02	-.05, .09	.03	0.54	.592
Gender × Perceived Partner Power	-.06	-.16, .04	-.07	-1.25	.213	-.02	-.10, .06	-.03	-0.56	.557
Gender × Actor Power × Perceived Partner Power	.02	-.07, .10	.02	0.42	.674	.03	-.04, .10	.05	0.94	.347

*Note*. Significant effects involving power are shown in bold. Gender = −1 = women, 1 = men. Study 2 involved dyadic multilevel regression models with *r* calculated as an indicator of effect size. Study 3 involved multiple regression analyses of individuals producing β as indicator of standardized effect size.

#### Sexual Accommodation–Neglect

As shown in Table 5, replicating Study 1, greater perceived partner power, but not actor’s power, predicted greater *willingness to compromise*. However, the results differed for enacted compromise: lower actor power rather than higher perceived partner power significantly predicted greater *enacted compromise*. As shown in Table 8, the results for *sexual communal strength* were as expected: greater perceived partner power, and not actor power, was associated with greater sexual communal strength. Thus, two of three measures of sexual accommodation–neglect supported predictions. Across measures, there were no significant Actor × Perceived Partner Power or Gender interactions.

**Table 8.** Associations Between Actor Power, Perceived Partner Power, and Sexual Communal Strength (Studies 2 and 3).

Predictors	*Sexual communal strength*				
	*b*	95% CI	*r/*β	*t*	*p*
*Study 2*					
Actor Power	.01	-.09, .12	.02	0.26	.795
Perceived Partner Power	**.23**	**.11, .35**	**.21**	**3.71**	**<.001**
Actor Power × Perceived Partner Power	-.04	-.15, .07	-.05	-0.78	.436
Gender	.21	.12, .31	.32	4.30	<.001
Gender × Actor Power	.09	-.02, .19	.10	1.68	.094
Gender × Perceived Partner Power	-.01	-.13, .11	-.01	-0.15	.885
Gender × Actor Power × Perceived Partner Power	.01	-.10, .11	.01	0.12	.907
*Study 3*					
Actor Power	.05	-.05, .15	.05	0.99	.322
Perceived Partner Power	**.34**	**.23, .45**	**.30**	**5.88**	**<.001**
Actor Power × Perceived Partner Power	.09	-.01, .18	.08	1.76	.079
Gender	.37	.29, .46	.39	8.26	<.001
Gender × Actor Power	.09	-.01, .19	.09	1.85	.065
Gender × Perceived Partner Power	-.08	-.20, .03	-.07	-1.46	.144
Gender × Actor Power × Perceived Partner Power	-.02	-.12, .08	-.02	-0.43	.665

*Note*. Significant effects involving power are shown in bold. Gender = −1 = women, 1 = men. Study 2 involved dyadic multilevel regression models with *r* calculated as an indicator of effect size. Study 3 involved multiple regression analyses of individuals producing β as indicator of standardized effect size.

#### Additional Analyses

Actor and partner power were positively associated with sexual satisfaction, but controlling for satisfaction did not alter the results, with one exception: the unexpected link between actor power and enacted compromise was no longer significant. In addition, controlling for dyadic sexual desire, which was not associated with actor or partner power, did not alter the results. See SM. Finally, we reran the analyses replacing perceived partner power with partner-reported power. Consistent with expectations that any effects of partner power should occur via perceptions of partner power, actor power continued to predict sexual approach–inhibition, but partner-reported power did not significantly predict accommodation–neglect (see SM for full results).

## Study 3

Study 2 largely supported predictions. Actors’ power predicted sexual approach–inhibition (sexual assertiveness–compliance) whereas perceived partner power generally predicted accommodation–neglect (willingness to compromise, communal strength). There were no significant Actor × Perceived Partner interactions, and only one gender difference: the link between actor power and sexual assertiveness was significant for men, not women. As expected, additional analyses revealed that perceived partner rather than partner-reported power predicted accommodation-neglect, which illustrates that accommodation emerges to sustain relationships with partners that actors perceive influence valued outcomes (see Overall et al., 2023).

 Only one finding was unexpected. Unlike Study 1, actor power rather than perceived partner power predicted enacted compromise. This pattern could indicate that high perceived partner power motivates partner accommodation as indicated by willingness to compromise and communal strength, but actual acts of compromise may involve partner accommodation (as suggested by Study 1) and inhibition, such as discomfort in refusing sex and compliance (linked to actor power in Studies 1 and 2). We re-examine these links in Study 3.

Study 3 included all measures used in Studies 1 and 2 for direct replication. Given Study 2 confirmed the principal role of perceptions of partner power in motivating accommodation–neglect, we sampled individuals rather than couples. To increase comparability with prior studies, we added another measure of compliance (labeled sexual acquiescence) previously used to investigate power and sexual compliance (Conroy et al., 2015; Himanen & Gunst, 2024). In addition, we measured responses to sexual rejection (Kim et al., 2019) to assess accommodation–neglect when it really matters—when actors want sex but their partner does not. We assessed understanding and acceptance of partners’ lack of desire for sex (sexual understanding), providing another indicator of accommodation. We also assessed attempts to coax partners by reinitiating sex and attempting to change their mind (sexual enticement), which directly neglects the partners’ stated desire. Our aim was to assess a more active, extreme form of partner neglect that might be particularly likely to occur in the context of perceived power asymmetries. In particular, sexual enticement represents actively ignoring the partner’s needs (extreme partner neglect that should be more likely when partners are low in power) to obtain or push actors’ own desires (extreme self-focused approach that could be more likely when actors are high in power). Thus, although Studies 1 and 2 provided no evidence that asymmetries were critical, we wanted to examine whether high power actors’ assertion of their needs allows for more harmful neglect of partners perceived low in power, as indicated by sexual enticement. We pre-registered predictions, research questions, and measurement decisions.

### Method

#### Participants and Procedure

Participants were compensated £5 for a 30-min questionnaire on Prolific.com. Participants were required to be sexually active, in committed man–woman relationships, childfree, and over 18. We sampled 450 participants (225 women, 225 men) to replicate the effects in Studies 1 and 2 with a larger sample, provide a reasonable sample for testing Actor × Perceived Partner Power asymmetries (80% power for small-median slopes of actor and perceived partner power in opposite directions requires *N* = 415; Sommet et al., 2023), and account for pre-registered exclusions; not involved in woman–man relationships (*n* = 26) or failed attention checks (*n* = 3). SM provides further information. Table 2 presents demographics of the final sample (211 women, 210 men). Participants were in well-established relationships (65% cohabiting or married). The majority resided in the United Kingdom or United States and identified as White/Caucasian.

#### Measures

##### Actor and Perceived Partner Power

The same scales in Studies 1 and 2 assessed actor (α = .86) and perceived partner (α = .82) power.

##### Sexual Approach–Inhibition

Participants rated the items assessing *Comfort in Initiating* and *Comfort in Refusing Sex* in Study 1 and completed the measures of *Sexual Assertiveness* (α = .92) and *Sexual Compliance* (α = .75) from Study 2. Participants also completed a measure used in prior studies examining power and sexual compliance called *Sexual Acquiescence* (Conroy et al., 2015; Himanen & Gunst, 2024). Unlike the items for the sexual compliance measure in Study 2, this scale distinguishes actors’ compliance from partners’ sexual coercion by asking participants to rate how often they participated in sexual activities they do not desire even though their partner does not pressure, threaten, or force them. Participants rated five sexual activities: “manual genital stimulation,” “oral intercourse,” “vaginal intercourse,” “anal intercourse,” and “other types of sexual activity” (e.g., “How often do you participate in, give, or receive vaginal intercourse that you do not desire?”). Response options: 0 = 0% (never), 1 = 25% (1 out of 4 times), 2 = 50% (about half the time), 3 = 75% (3 out of 4 times), 4 = 100% (all the time). Items were averaged (α = .84). General reports of engaging in undesired sexual activity (sexual compliance) and reported frequencies of engaging in specific undesired activities (sexual acquiescence) were moderately correlated (*r* = .46), indicating conceptual overlap along with distinctions in assessing sexual compliance.

##### Sexual Accommodation–Neglect

Participants rated the items assessing *Enacted Compromise* and *Willingness to Compromise* in Studies 1 and 2. Participants selecting “not applicable” (16 for enacted, 3 for willingness) slightly reduced the sample for analyses. Participants also completed the *Sexual Communal Strength* measure (α = .78) from Study 2.

Finally, participants completed the responses to sexual rejection scale (Kim et al., 2019). Participants were asked: “Please rate how frequently you tend to react in the following ways when you want to have sex but your partner lets you know that they do not.” Three items assessed *Sexual Understanding* (e.g., When I want to have sex, but my partner lets me know that they do not . . . “I let my partner know I still love them,” “I am understanding and accepting”; 1 = *never*, 5 = *very frequently*”; α = .73). Four items assessed *Sexual Enticement* (e.g., “I continue trying to convince my partner to have sex”; “I try initiating sex with my partner again”; α = .83), which directly neglects partner’s needs by ignoring their stated desire to not have sex to pursue actors’ own desires.

##### Additional Analyses Measures

Participants completed the sexual satisfaction measure from Study 1 and a three-item version of dyadic sexual desire from Study 2 (see SM).

### Results

Table 3 presents descriptives (SM presents correlations). As in Study 1, we regressed each outcome on: actor power, partner power, the Actor × Perceived Partner Power interaction, gender, and all gender interactions.

#### Sexual Approach–Inhibition

Results for *comfort initiating sex* and *comfort refusing sex* replicated Study 1 (Table 4): Actor power (and not perceived partner power) positively predicted both variables. As in Study 1, the Actor × Perceived Partner Power interaction also was significant when predicting comfort refusing sex, but significant gender differences revealed this interaction was only significant for men (*b* = .45, 95% CI = [.17, .73], β = .22, *t* = 3.13, *p* = .002), not women (*b* = .04, 95% CI = [−.21, .27], β = .02, *t* = 0.28, *p* = .777). For men, actor power significantly predicted comfort refusing sex only when perceived partner power was high (+1 *SD: b* = .70, *t* = 3.65, *p* < .001) but not low (−1 *SD: b* = −.07, *t* = −0.36, *p* = .718) indicating that low perceived partner power stifles rather than amplifies the sexual assertiveness of high power men (see SM for details).

Results for *sexual assertiveness* (Table 6) supported predictions: actor power positively predicted assertiveness. Unlike Study 2, this link did not differ by gender and the Actor × Perceived Partner Power interaction was significant. The interaction aligned with that for comfort refusing sex: actor power more strongly predicted assertiveness when perceived partner power was high (+1 *SD: b* = .33, *t* = 6.50, *p* < .001) versus low (−1 *SD: b* = .15, *t* = 2.81, *p* = .005; see SM).

The results for *sexual compliance* and *sexual acquiescence* generally replicated the results for compliance in Study 2 (Table 7): lower actor power was significantly associated with higher compliance and acquiescence. Unexpectedly, lower perceived partner power also predicted higher sexual compliance (but not acquiescence).

#### Sexual Accommodation–Neglect

The results for sexual compromise (Table 5) revealed that, as in Study 2, lower actor power and not higher partner power predicted greater *enacted compromise*. Replicating Study 1 and 2, however, greater perceived partner power predicted greater *willingness to compromise.* Unexpectedly, gender differences emerged for actor power and the Actor × Perceived Partner Power interaction. For men, higher actor power also predicted greater willingness to compromise (*b* = .22, 95% CI = [.02, .41], β = .17, *t* = 2.19, *p* = .029) but only when perceived partner power was low (−1 *SD: b* = .35, *t* = 2.70, *p* = .008) not high (+1 *SD: b* = .08, *t* = .66, *p* = .508). For women, only perceived partner and not actor power predicted willingness to compromise (see SM). Thus, both women and men were more willing to compromise for partners perceived high in power, but high power men also were willing to compromise for partners they perceived low in power.

The results for *sexual communal strength* replicated Study 2 (Table 8): higher perceived partner power, and not actor power, predicted higher communal strength. Similarly, the results for *sexual understanding* (Table 9) revealed that higher perceived partner power, and not actor power, predicted greater sexual understanding. The Actor × Perceived Partner Power interaction also was significant: the positive link between perceived partner power and sexual understanding was significant when actor power was high (+1 *SD: b* = .38, *t* = 4.27, *p* < .001) but not low (−1 *SD: b* = .02, *t* = 0.29, *p* = .769) such that sexual understanding was greatest when both actor and perceived partner power were high (see SM).

**Table 9.** Associations Between Actor Power, Perceived Partner Power, and Sexual Understanding and Enticement (Study 3).

Predictors	*Sexual understanding*					*Sexual enticement*				
	*b*	95% CI	*r/*β	*t*	*p*	*b*	95% CI	β	*t*	*p*
*Study 3*										
Actor Power	.01	-.10, .12	.01	0.18	.859	-.02	-.12, .08	-.02	-0.37	.712
Perceived Partner Power	**.20**	**.07, .33**	**.17**	**3.05**	**.002**	-.10	-.22, .02	-.09	-1.60	.110
Actor Power × Perceived Partner Power	**.20**	**.09, .31**	**.17**	**3.50**	**<.001**	.08	-.02, .18	.07	1.53	.127
Gender	.00	-.10, .11	.01	0.07	.944	.23	.13, .32	.25	4.71	<.001
Gender × Actor Power	-.04	-.15, .07	-.04	-0.72	.471	.03	-.07, .14	.03	0.63	.530
Gender × Perceived Partner Power	.03	-.10, .16	.02	0.39	.697	-.07	-.19, .05	-.06	-1.13	.261
Gender × Actor Power × Perceived Partner Power	.01	-.10, .12	.01	0.23	.822	-.04	-.14, .07	-.04	-0.68	.495

*Note*. Significant effects involving power are shown in bold. Gender = −1 = women, 1 = men.

Finally, there were no significant associations between power and *sexual enticement* (Table 9), and although men reported higher enticement, gender did not moderate the effects of power.

#### Additional Analyses

Actor and perceived partner power were positively associated with sexual satisfaction and desire, but controlling for these variables did not alter the significant associations in Study 3 with one exception: the Actor × Perceived Partner Power interaction for sexual assertiveness was no longer significant when controlling sexual satisfaction (see SM).

## Discussion

The current studies show that actor and perceived partner power differentially relate to sexual behaviors and motivations. Actors’ power related to sexual approach–inhibition: actors high in power reported more comfort initiating and refusing sex, more assertive communication about sex, and less compliance to undesired sexual activity (see Table 1). Perceived partner power related to sexual accommodation–neglect: when partners were perceived to be high in power, actors expressed greater willingness to compromise, sexual communal strength, and understanding when partners rejected sex. These distinct effects were not magnified by asymmetries between actor and perceived partner power, nor did they differ across gender. Only two results diverged from predictions (see Table 1): low actor power rather than high perceived partner power predicted greater enacted sexual compromise, and perceived partner power (or actor power) was not significantly associated with sexual enticement. The expected and unexpected results advance new insights into how power can promote harmful and beneficial behavior.

### New Insights: Actor Power and Perceived Partner Power Relate to Distinct Sexual Behaviors and Motivations

Distinguishing the effects of actor versus perceived partner power clarifies the theoretical processes most relevant to distinct behaviors and motivations. We illustrate with sexual approach–inhibition. Consistent with our results, prior studies have applied similar principles to specify that high power actors will confidently assert their sexual desires (e.g., Birnbaum et al., 2025; Lammers & Stoker, 2019). However, prior studies have implied that sexual compliance—engaging in undesired sexual activity—emerges from perceiving partners have higher power, and is most often motivated by promoting intimacy or partners’ pleasure (e.g., Conroy et al., 2015; Himanen & Gunst, 2024). Our results challenge this view. Lower actor power and not higher perceived partner power predicted greater sexual compliance, revealing that compliance will often reflect inhibition to prevent negative consequences that low power actors cannot control rather than motivation to accommodate the desires of partners perceived high in power. This distinction has important implications. Sexual compliance arising from low confidence and avoiding negative consequences is more harmful than compliance motivated to enhance intimacy with or care for partners (Himanen & Gunst, 2024; Katz & Tirone, 2009; Nickull et al., 2025; see Impett & Peplau, 2003).

Assessing a range of behaviors and motivations also provides deeper insight into the way power shapes behavior. Most investigations focus on how power affects specific behaviors (e.g., assertiveness or compliance) rather than examining various outcomes relevant to different theories and psychological processes (i.e., approach–inhibition vs. accommodation–neglect). The current studies provide the first illustration that actors are motivated to accommodate the sexual needs and preferences of partners perceived high in power, as shown by greater willingness to compromise and sexual communal strength. These motivations generate more responsive behavior and sustain satisfying relationships when couples face sexual conflicts or difficulties (e.g., Day et al., 2015; Muise, Bergeron, et al., 2017), including understanding when partners are not in the mood (as shown by our results). Critically, these links were distinct from actor power and inhibition (low assertiveness, greater compliance) showing that accommodating partners’ needs—and associated relational benefits—does not inherently entail actors inhibiting their own needs and desires.

The importance of disentangling accommodation–neglect from approach–inhibition is emphasized by the unexpected results for enacted compromise. High perceived partner power predicted greater willingness to compromise in all studies, but low actor power predicted enacted compromise in two studies. Our measures of compromise did not differentiate whether compromises involved accepting partners wishes to not engage in sex as frequently or in the way actors desired (accommodation) or agreeing to engage in sex more frequently or in the way partners wish despite actors desiring otherwise (inhibition). Enacted compromise may have captured the latter given the pattern across outcomes: high perceived partner power predicted actors accommodating their partner’s lack of desire (e.g., sexual understanding) whereas low actor power predicted actors engaging in undesired activity that their partner desired (e.g., sexual compliance). Our findings highlight the need to differentiate two distinct types of sexual compromise: (a) accommodating partners’ preference to *not* engage in sexual activities that actors desire versus (b) actors inhibiting their own preferences to engage in undesired sexual activities that their partner desires.

By teasing apart the effects of actor and perceived partner power, the combined results emphasize that having power is more beneficial than harmful. Some perspectives warn that power can be dangerous by providing the opportunity to exploit low power others (Galinsky et al., 2015). Yet, the assertiveness related to actors’ high power and accommodation related to perceiving partners’ high power promotes satisfying sex lives for both actors and partners (Couture et al., 2025; Impett et al., 2020; Zhang et al., 2022). Moreover, high actor power did not produce poor treatment of partners. High actor power promoted greater assertiveness, but did not predict less willingness to compromise, communal strength, or understanding. Moreover, low perceived partner power predicted partner neglect (less willingness to compromise, communal strength, understanding), but did not prompt actors’ continued pursuit of sexual desires that disregarded partners’ wishes because low perceived partner power (or high actor power) did not significantly predict sexual enticement. And, as above, high perceived partner power promoted accommodation but did not compel inhibition and compliance.

Tests of interactions between actor and perceived partner power also support that having or perceiving power has dyadic benefits. Although power is often conceptualized in relative terms, asymmetries did not play a key role, including no evidence that low actor/high perceived partner power prompted compliance or high actor/low perceived partner power prompted neglect. Instead, the few significant Actor × Perceived Partner Power interactions indicated that both actors and partners having power is sexy. Three interactions indicated high actor power promoted greater sexual assertiveness, which was stifled when partners were perceived to have low power. Two interactions revealed accommodation was greatest when actor and perceived partner power were both high, and another suggested high power men also were accommodating of partners perceived low in power. Although we express caution in interpreting six unpredicted interactions from 19 tests (see SM), the overall findings underscore that jointly holding perceived power is beneficial rather than asymmetries in perceived power being harmful.

The results also illustrate that perceived power equally shapes women’s and men’s sexual behavior. Gender differences in levels of behavior tended to follow traditional sexual scripts, such as women reporting lower sexual assertiveness and higher sexual compliance. Yet, the effects of power did not systematically vary across men and women (three gender differences in 19 tests, none of which replicated across studies). Other studies also have found no gender differences in the effects of actor or perceived partner power on non-sexual (e.g., Overall et al., 2023; Righetti et al., 2015) and sexual (e.g., Birnbaum et al., 2025; Lammers & Stoker, 2019) behavior. Thus, investigations only including women overlook that facilitating both women’s and men’s perceived power in intimate relationships should reduce sexual compliance and neglect and promote assertiveness and accommodation of women’s and men’s sexual needs.

### Caveats and Future Directions

Correlational data prevent causal conclusions. Our findings join experimental demonstrations that power prompts approach–inhibition or accommodation–neglect (Keltner et al., 2003; Pike & Galinsky, 2020), but reverse directions and reinforcing loops are possible. Greater inhibition may reinforce actors’ low power and accommodation could affirm perceptions of partners’ power. Moreover, some behaviors may intentionally regulate power. For example, partners’ perceived controlling behavior predicts women’s compliance (Conroy et al., 2015; Himanen & Gunst, 2024). Rather than reflecting partners’ high power, partners’ controlling or coercive behavior is most likely motivated by partners feeling they have lost power (Overall & Hammond, 2026), but has the intended consequence of down-regulating actors’ felt power and promoting compliance (Tirone & Katz, 2020). Future experimental and longitudinal studies could provide stronger evidence for the behaviors that emerge from and shift perceived power.

Like prior studies examining power and sexual behavior, we used self-report measures. Self-reports of behaviors do not clearly assess intentions or awareness. For example, low compromise may reflect neglecting partners’ needs by failing to care for and support partners or by actively ignoring partners’ desires. Null links with sexual enticement suggest that low perceived partner power promotes actors failing to support rather than actively disregarding partners’ needs. Gathering implicit measures and/or partner reports of behavior that actors may under-report, such as sexual enticement, could strengthen future tests. Critically, however, actors’ perceptions are central to understanding power. As in prior studies, the effects of partner power emerged from actors’ perceptions of partner power rather than partner-reported power (Study 2). Perceptions of partner power should be most critical if accommodation-neglect emerges from dependence on partners that actors perceive control valued outcomes (Kelley & Thibaut, 1978; Overall et al., 2023). Alternatively, partner-reported power should have equal or greater effects if accommodation emerges because high power partners force their preferences (see Overall et al., 2023). None of our results support this alternative.

Our multi-assessment approach allowed us to replicate distinct effects of actor and partner power using measures that may capture distinct types or severity of sexual behaviors. For example, our conceptualization of sexual compliance—consenting to or engaging in undesired sexual activity—aligned with prior studies (e.g., Conroy et al., 2015; Himanen & Gunst, 2024; Katz & Tirone, 2009; Nickull et al., 2025), but prior studies have assessed compliance in slightly different ways. Unlike prior studies, we replicated effects across two different measures that assessed engaging in unwanted sex (e.g., “I find myself having sex when l do not really want it”; Couture et al., 2025; Hurlbert, 1991) and acquiescence to participate in sexual activities that participants did not desire even though their partner did not pressure, threaten, or force them (Conroy et al., 2015; Himanen & Gunst, 2024). It is possible these two measures capture different types of compliance and/or they may assess more negative or extreme compliance than other measures that target engaging in sexual activity without initial sexual desire (e.g., Nickull et al., 2025). Yet, lower actor power was significantly associated with higher compliance (unwanted sex) and acquiescence (sexual activities without desire) indicating they both involve inhibition. Moreover, if our measures captured particularly negative forms of compliance then our findings emphasize the important consequences of low power and sexual inhibition. Future studies that also assess multiple measures are needed to assess the existence, predictors, and implications of distinct types of compliance (and other inhibition behaviors).

Our samples may have been underpowered to detect small Actor × Perceived Partner Power interactions (Sommet et al., 2023). Yet, if asymmetries are crucial, then actor or perceived partner power on their own would be unreliable predictors or show opposing effects on the same behavior (e.g., actor power negatively and perceived partner power positively predicting compliance). Instead, actor and perceived partner power exerted reliable independent effects on distinct behaviors. This pattern emphasizes that actor and perceived partner power have important behavioral implications across all types of relationships, not only those characterized by asymmetries. Importantly, null interactions do not mean that actor and partner power combinations are irrelevant. Additive combinations of distinct effects means that the best couple outcomes will emerge when high actor + high perceived partner power leads actors to approach their needs while also accommodating their partner’s needs. By contrast, low actor power + low perceived partner power will involve actors’ inhibiting their needs while neglecting their partner’s needs, and high actor + low perceived partner power will involve actors’ approaching their needs while neglecting their partner’s needs, both risking unhealthy relationships (see Overall & Hammond, 2026).

Our investigation examined power and sexual behavior within long-term intimate relationships characterized by high interdependence—actors and partners care about and have some power over each other. In non-intimate contexts, larger asymmetries may be more influential; one person holding power over another who has little or no counter-power could afford particularly harmful inhibition and neglect behavior. Even within intimate relationships, asymmetries may play a stronger role in couples characterized by conflict, dissatisfaction, and power struggles that are unlikely to participate in relationship studies and thus not represented in our samples. Moreover, in younger samples and dating contexts devoid of longer-term commitment, perceived power may be more tenuous and influenced by broader societal power differentials that govern gender roles and scripts (Conroy et al., 2015). Wider power differentials also will limit how power is expressed and responded to in intimate relationships (Overall & Hammond, 2026), and thus gender differences and asymmetries in perceived power may be more apparent within countries with greater gender inequality or cultural norms that restrict sexual behavior. All of these power-restraining contexts may allow for actors’ motives for power to override motives to care for partners resulting in more neglect, coaxing, coercion, and objectification. Applying the theoretical principles in Table 1 to these contexts will advance understanding of the benefits and potential dangers of actor and perceived partner power.

### Conclusions

Healthy sexual relationships involve actors being able to satisfy their own desires while accommodating their partner’s needs and desires. Hitting this sweet spot involves both actors and partners having perceived power. By applying, integrating, and advancing different theories of power, the current studies illustrate that actors’ power promotes sexual approach versus inhibition, including more sexual assertiveness and less compliance, and perceptions of partners’ power promotes sexual accommodation versus neglect, including greater willingness to compromise, sexual communal strength, and sexual understanding. These distinct effects were not magnified by asymmetries in actor and perceived partner power, nor did they differ across gender. Facilitating both actor and perceived partner power in intimate relationships should reduce rather than intensify the risk of harmful sexual behavior.

## Supplemental Material

sj-docx-1-psp-10.1177_01461672251390006 – Supplemental material for Actor Power and Perceived Partner Power Differentially Relate to Sexual Behavior and Motivations Supplemental material, sj-docx-1-psp-10.1177_01461672251390006 for Actor Power and Perceived Partner Power Differentially Relate to Sexual Behavior and Motivations by Nickola C. Overall, Jessica A. Maxwell, Amy Muise, Nina Waddell and Auguste G. Harrington in Personality and Social Psychology Bulletin
