# Supplementary material for: Actor Power and Perceived Partner Power Differentially Relate to Sexual Behavior and Motivations
Source: Pers Soc Psychol Bull. 2025 Nov 27;52(11):3904–23. doi: 10.1177/01461672251390006 (PMC13646409; doi:10.1177/01461672251390006)
Supplement: Supplementary file 1 [file sj-docx-1-psp-10.1177_01461672251390006.docx]

**Actor and Perceived Partner Power Differentially Relate to**

**Sexual Behavior and Motivations**

**Supplemental Material**

**Table of Contents**

Additional Sample Information and Statistical Power (Studies 1-3)	2

Measures (Studies 1-3)	5

Additional Information and Analyses for Measure of Compromise (Studies 1 and 3)	12

Correlations Across Variables (Studies 1-3)	14

Further Details and Figures of Actor X Perceived Partner Power Interactions (Studies 1-3)	16

Summary of Additional Analyses Measures and Results (Studies 1-3)	21

Femininity and Masculinity Ideology (Study 1)	21

Sexual Satisfaction (Studies 1-3)	22

Dyadic Sexual Desire (Studies 2 and 3)	22

Partner Reported Power (Study 2)	23

References	27

**Additional Sample Information and Statistical Power (Studies 1-3)**

**Study 1**

The data for Study 1 was drawn from an existing dataset. The online questionnaire was completed in February 2020. To be eligible, participants were required to be sexually active, in committed man-woman romantic relationships, childfree, and over the age of 18. The broader study required that participants were working or studying; however, because this criterion is irrelevant to the present hypotheses, we retained 6 participants who were not working or studying. Given the focus on power and sexual behavior within man-woman relationships, we included participants who identified as heterosexual and identified as either a man or woman. Participants were asked “What is the gender with which you identify?” and responded to the options ‘female’, ‘male’, and ‘gender diverse (specify if you wish)’. We recognize female and male are more indicative of biological sex than gender identity. Given we asked participants to indicate their gender identity, we refer to women and men participants in the paper. The final sample size for analyses was 270 participants (130 women, 140 men).

The sample size for this study was determined by practical constraints (e.g., funding) with the intention to detect small-medium effects. We conducted a post-hoc sensitivity analysis using G*Power (Faul et al., 2009) to determine what effect size we were powered to detect in the primary analyses reported in the paper. We conducted a sensitivity test for a linear multiple regression: fixed model, single regression coefficient, specifying 7 predictors, 80% power, and a sample size of 270. The results indicated we were powered to detect a significant effect of *f*^2^ = .029 (see screenshot below), which is considered a small effect (Cohen, 1988) and translates to a *β/r* of 0.17. The significant effects of actor and partner power in Study 1 were greater than *β/r* = .17 (≥.21).

One prior paper has used these data to examine associations between femininity and masculinity ideology and comfort in initiating and rejecting sex (Harrington & Maxwell, 2023), but no prior papers have examined the links between power, comfort in initiating and rejecting sex, or sexual compromise, as examined in the current study. Additional analyses also revealed that the current results were independent of femininity and masculinity ideology (see Additional Analyses, Femininity and Masculinity Ideology, below).

**Study 2**

Prior to pre-registration, data had been collected as part of a larger study, which included 176 couples completing questionnaires online or during an in-person research session. Data collection occurred from February 2021 to July 2024. The original target was for 200 couples but pandemic-related disruptions that delayed the beginning of data collection and created disruptions because of lockdowns and restrictions during the data collection period meant we did not reach our target by the time we had to end data collection in July 2024 due to funding timelines and associated staffing constraints. The eligibility criteria for participation included sexually active couples in committed, cohabiting romantic relationships of 6 months or longer, who were both aged 18 and above, either employed or enrolled in university studies, and did not have children. As in Study 1, we focus only on woman-man couples because prior theory and research suggest that the links between power and sexual behavior may be gendered, particularly within heterosexual contexts, and thus was planned to test for gender differences across distinguishable dyads.

The sample for analysis includes 152 man-woman couples (N = 304). We assessed achieved power to detect the smallest effect size of actor and perceived partner power in Study 1 (*β/r* = .21) based on an actor-partner interdependence model to account for the dependence between partners (α = .05; correlation between errors = .30; correlations between actor and partner power = .29, Ackerman et al., 2020) assuming, as in Study 1, there were no gender differences. We had high statistical power (.97) to detect the smallest effect size of actor and perceived partner power in Study 1 (see screenshot below).

Each individual was compensated NZ$40 in vouchers for both components of the study. The measures for the current study were completed during the in-person session. The measures across the study assessed a variety of constructs including individual differences (e.g., attachment, personality), relationship processes and outcomes (e.g., commitment, problems, support), and experiences in a range of life domains, such as work (e.g., burnout, work-life conflict), personal wellbeing (e.g., mental health, stress), and sex. We pre-registered our focus on the measures we reported to align with those assessed in Study 1 and our specific predictions regarding the links between power and sexual approach–inhibition and sexual accommodation–neglect. No papers have been published using data from the broader study.

**Study 3**

The data for Study 3 was designed and collected specifically to replicate the associations in Studies 1 and 2. Mimicking the procedures in Study 1, the online questionnaire was completed in November 2024. As we pre-registered , we aimed to collect a useable sample size of 400 participants (200 women, 200 men) to replicate effects found in prior samples (Study 1, N = 270; Study 2, N = 304), test for gender differences, and provide a reasonable sample for testing interactions between actor and partner power that involve asymmetries in power (i.e., small-medium slopes of actor and partner power in opposite directions; Sommet et al., 2023).

The final sample of 421 participants exceeds Studies 1 and 2 which were already powered for the smallest effect size found in Studies 1 and 2 (as reported above). We followed Sommet et al.’s (2023) guidelines for assessing sample size for the type of interaction that should emerge if power asymmetries are crucial. A sample of 421 achieves 80% power for a reverse interaction involving a small (.20) and median (.35) effect in opposite directions (see table below from Sommet et al., 2023). Please see Discussion for further consideration of power for actor X partner interactions.

**Measures** **(Studies 1-3)**

**Actor and Perceived Partner Power: Studies 1-3**

In all three studies, participants completed the 8-item relationship-specific Sense of Power Scale (Anderson et al., 2012) to assess their own power (actor power) and perceptions of their partner’s power (perceived partner power).

*Instructions:* Please indicate the degree to which you agree with each of the following statements regarding your CURRENT ROMANTIC RELATIONSHIP.

*Response option 7-point Likert-type scale:* 1 = strongly disagree, 7 = strongly agree.

***Actor Power***

In my relationship …

I can get my partner to listen to what I say.

My wishes do not carry much weight.

I can get my partner to do what I want.

Even if I voice them, my views have little sway.

I think I have a great deal of power.

My ideas and opinions are often ignored.

Even when I try, I am not able to get my way.

If I want to, I get to make the decisions.

***Perceived Partner Power***

In my relationship …

My PARTNER can get me to listen to what he/she says.

My PARTNER’S wishes don’t carry much weight.

My PARTNER can get me to do what he/she wants.

Even if MY PARTNER voices their views, his/her views have little sway.

I think my PARTNER has a great deal of power.

My PARTNER’S ideas and opinions are often ignored.

Even when my PARTNER tries, he/she is not able to get his/her way.

If my PARTNER wants to, he/she gets to make the decisions.

**Approach-Inhibition Measures**

***Comfort Initiating* and *Comfort Refusing Sex: Studies 1 and 3***

*Instructions:* Please rate your agreement/disagreement with the following items.

*Response option 7-point Likert-type scale:* 1 = strongly disagree, 7 = strongly agree.

I am comfortable initiating sex.

I am comfortable refusing sex.

***Sexual Assertiveness and Compliance: Studies 2 and 3***

Participants completed the 25-item Hurlbert Index of Sexual Assertiveness (Hurlbert, 1991) to assess *sexual assertiveness* (16 items) and *sexual compliance* (3 items).

*Instructions:* This question asks about your views on sex in general. Please respond to the following items using the scale provided.

*Response option 5-point scale:* 0 = All of the time, 1 = Most of the time, 2 = Some of the time, 3 = Rarely, 4 = Never.

*Sexual Assertiveness*

I feel uncomfortable talking during sex.

I feel that I am shy when it comes to sex.

I approach my partner for sex when I desire it.

I think I am open with my partner about my sexual needs.

I enjoy sharing my sexual fantasies with my partner.

I communicate my sexual desires to my partner.

It is difficult for me to touch myself during sex.

I feel uncomfortable telling my partner what feels good.

I speak up for my sexual feelings.

When a technique does not feel good, I tell my partner.

I feel comfortable giving sexual praise to my partner.

It is easy for me to discuss sex with my partner.

I feel comfortable in initiating sex with my partner.

I feel comfortable telling my partner how to touch me.

It is hard for me to be honest about my sexual feelings.

I try to avoid discussing the subject of sex.

*Sexual Compliance*

I find myself having sex when I do not really want it.

I find myself doing sexual things that I do not like.

It is hard for me to say no even when I do not want sex.

*Items Assessing Other Constructs Excluded in Study 2 and Not Collected in Study 3*

I enjoy masturbating myself to orgasm. *(not about the partner)*

I feel uncomfortable talking to my friends about sex. *(not about the partner)*

I am reluctant to describe myself as a sexual person. *(not about the partner)*

If something feels good, I insist on doing it again. *(insistence not assertiveness)*

I am reluctant to insist that my partner satisfy me. *(insistence not assertiveness)*

Pleasing my partner is more important than my pleasure. *(unmitigated communion)*

***Sexual Acquiescence: Study 3***

Participants completed a measure of sexual acquiescence used in prior studies assessing the links between power and sexual compliance (Conroy et al., 2015; Himanen & Gunst, 2022).

*Instructions:* How often do you participate in the following activities even though your partner does NOT pressure, threaten, or force you?

*Response option 5-point scale:* 0 = 0% (never), 1 = 25% (1 out of 4 times), 2 = 50% (about half the time), 3 = 75% (3 out of 4 times), and 4 = 100% (all the time).

How often do you participate in, give, or receive manual genital stimulation that you do not desire?

How often do you participate in, give, or receive vaginal intercourse that you do not desire?

How often do you participate in, give, or receive oral intercourse that you do not desire?

How often do you participate in, give, or receive anal intercourse that you do not desire?

How often do you participate in, give, or receive other types of sexual activity that you do not desire?

**Accommodation-Neglect Measures**

***Sexual Compromise: Studies 1-3***

Participants completed new scales assessing frequency of compromise and willingness to comprise. Some of these items were inspired by past research on ‘sexual transformations’ (Burke & Young, 2012) or ‘sexual accommodations’ (Maxwell et al., 2017). Items were presented in random order.

*Note.* The primary analyses used the items presented below. See information about other items and supplemental analyses in the following section of this document.

*Enacted Compromise*

*Instructions:* Over the PAST SIX MONTHS, please indicate how frequently you have made compromises with your partner in terms of:

*Response option 7-point Likert-type scale:* 1 = never compromised, 7 = regularly compromised; with additional option to indicate “not applicable”.

Frequency of sexual activity.

Types of sexual activities engaged in (e.g., oral sex, sexting).

*Willingness to Compromise*

*Instructions:* How willing would you be to make compromises with your partner in terms of:

*Response option 7-point Likert-type scale:* 1 = not at all willing, 7 = extremely willing; with additional option to indicate “not applicable”.

Frequency of sexual activity.

Types of sexual activities engaged in (e.g., oral sex, sexting).

***Sexual Communal Strength: Studies 2 and 3***

Participants completed the sexual communal strength measure developed by Muise et al. (2013). The original measure included 6 items, but in previous research (e.g., Balzarini et al., 2021), the two reverse coded items loaded onto a distinct factor and the resulting 4-item measure was reliable. We used this four-item version.

*Instructions:* Please indicate the extent to which you agree with each of the following statements regarding your current romantic relationship.

*Response option 7-point Likert-type scale:* 1 = not at all, 7 = extremely.

How far would you be willing to go to meet your partner's sexual needs?

How high a priority for you is meeting the sexual needs of your partner?

How easily could you accept not meeting your partner's sexual needs?

How happy do you feel when satisfying your partner's sexual needs?

***Sexual Understanding and Enticement: Studies 1 and 2***

Participants completed the Responses to Sexual Rejection scale (Kim et al., 2020).

*Instructions:* Please rate how frequently you tend to react in the following ways when you want to have sex but your partner lets you know that they do not?

When I want to have sex, but my partner lets me know that they do not ...

*Response option 5-point Likert scale:* 1 = Never, 5 = Very frequently.

*Sexual Understanding*

I let my partner know I still love them.

I let my partner know I am still attracted to them.

I am understanding and accepting.

I try not to express negative emotions.

*Note.* The last item did not perform well (item-total correlation = .125, α = .60) and so was excluded. The other three items were averaged to index sexual understanding (α = .73).

*Sexual Enticement*

I attempt to change my partner's mind (e.g., try to tempt or seduce them).

I continue trying to convince my partner to have sex.

I try initiating sex with my partner again.

I ask if there is anything I can do to get my partner in the mood.

**Additional Analyses Measures**

***Sexual Satisfaction: Studies 1 and 3***

Five items from the Global Measure of Sexual Satisfaction (Lawrance & Byers, 1995).

*Instructions:* Please rate your sex life on the following dimensions.

*Items in semantic differential format:*

Bad ○ ○ ○ ○ ○ ○ ○ Good

Unpleasant ○ ○ ○ ○ ○ ○ ○ Pleasant

Negative ○ ○ ○ ○ ○ ○ ○ Positive

Unsatisfying ○ ○ ○ ○ ○ ○ ○ Satisfying

Worthless ○ ○ ○ ○ ○ ○ ○ Valuable

***Sexual Satisfaction: Study 2***

Participants completed the Quality of Sex Scale (Shaw & Rogge, 2016), which contains two subscales (12 items each) that assess *sexual satisfaction* and *sexual dissatisfaction*.

*Instructions:* Please indicate the extent to which you agree with each of the following statements regarding your current romantic relationship.

*Response option 6-point scale:* 0 = Not at all true, 1 = A little true, 2 = Somewhat true, 3 = Mostly true, 4 = Very true, 5 = Completely true.

*Sexual Satisfaction*

My sex life is fulfilling.

I am happy with my sex life with my partner.

My partner really pleases me sexually.

I am satisfied with our sexual relationship.

I am happy with the quality of sexual activity in our relationship.

Sexual activity with my partner is fantastic.

I am happy with my partner as a lover.

Sexual activity with my partner is rewarding.

Sexual activity with my partner is enjoyable.

My sex life is very exciting.

Sexual activity with my partner is everything I could hope for.

Sex is fun for my partner and me.

*Sexual Dissatisfaction*

Sexual activity with my partner is not fun.

Sexual activity with my partner is a turn off.

Sexual activity with my partner is not worth the time or effort.

I do not enjoy sexual activity with my partner.

Sexual activity with my partner leaves me empty.

Sexual activity with my partner is not very exciting.

I would rather not engage in sexual activity with partner.

I don't look forward to sexual activity with my partner.

My sex life with my partner has become somewhat dull.

I am tired of engaging in sexual activity with my partner.

Sexual activity with my partner leaves me feeling distant and alone.

I am very disappointed with my sex life with my partner.

***Dyadic Sexual Desire: Study 2***

Participants completed the Sexual Desire Inventory (Spector et al., 1996). Eight items assessed desire to engage in sexual activity with a partner.

*Instructions:* Please indicate the extent to which you agree with each of the following statements regarding your current romantic relationship. During the last month, how often...

*Response option 7-point Likert-type scale:* 1 = not at all, 7 = very often.

Would you have liked to engage in sexual activity with a partner (for example, touching each other's genitals, giving or receiving oral stimulation, intercourse, etc)?

*Instructions:* Please indicate the extent to which you agree with each of the following statements.

*Response option 7-point Likert-type scale:* 1 = not strong at all, 7 = very strong.

When you have sexual thoughts, how strong is your desire to engage in sexual behaviour with a partner?

When you first see an attractive person, how strong is your sexual desire?

When you spend time with an attractive person (for example, at work or school), how strong is your sexual desire?

When you are in romantic situations (such as a candlelit dinner, a walk on the beach, etc), how strong is your sexual desire?

How strong is your desire to engage in sexual activity with a partner?

How important is it for you to fulfil your sexual desire through activity with a partner?

***Dyadic Sexual Desire: Study 3***

Participants completed three times from the Sexual Desire Inventory (Spector et al., 1996) which represented the top loading items specifically referring to sexual desire toward a partner in prior factor analyses of the sexual desire inventory (Moyano et al., 2017).

*Instructions:* Please indicate the extent to which you agree with each of the following statements.

*Response option 7-point Likert-type scale:* 1 = not strong at all, 7 = very strong.

When you have sexual thoughts, how strong is your desire to engage in sexual behaviour with a partner?

How strong is your desire to engage in sexual activity with a partner?

How important is it for you to fulfil your sexual desire through activity with a partner?

**Additional Information and Analyses for Measure of Compromise (Studies 1 and 3)**

In Study 1, participants completed new scales assessing sexual compromise, including 12 items. Some of these items were inspired by past research on ‘sexual transformations’ (Burke & Young, 2012) or ‘sexual accommodations’ (Maxwell et al., 2017). For *enacted compromise*, participants were asked “Over the PAST SIX MONTHS, please indicate how frequently you have made compromises with your partner in terms of …” and for *willingness to compromise*, participants were asked “How willing would you be to make compromises with your partner in terms of …”.

Frequency of sexual activity

Types of sexual activities engaged in (e.g., oral sex, sexting)

Use of sex toys

Role playing

Sexual positions

Sexual attire (e.g., lingerie)

Location of sex

Duration of sexual activities (e.g., duration of foreplay)

Time of day of sex

Way we initiate sex

Sexual role taken (e.g., dominant/submissive)

Way we say no to/decline sex

For the purposes of our investigation, we selected two items that we reasoned should be related to power dynamics in woman-man relationships: items assessing compromise in (a) frequency of sexual activity, and (b) types of sexual activities engaged in (e.g., oral sex, sexting). These are two aspects of the sex lives of mixed-gender couples that are likely to be experienced regularly (e.g., MacNeil & Byers, 1997) compared to compromises that may be relevant to smaller proportions of couples (e.g., sex toys, role playing). Thus, in Study 1 we tested the predicted associations with the average of these two items. In Studies 2 and 3, our pre-registered analyses also only focused on these two items.

To be fully transparent, we also present the analyses with scores averaging all 12 items for enacted compromise and willingness to compromise (see Table SM1 on following page). The significant associations were the same across studies, with one exception: the association between partner power and enacted compromise in Study 1 was not significant with the full scale. This inconsistency aligns with the associations found in Studies 2 and 3 in which actor power, not partner power, was associated with enacted compromise, and which replicated with the full scale (see Table SM1). Please see our discussion in the paper regarding our interpretation of the unexpected association between actor power and enacted compromise (compared to the association between partner power and willingness to compromise).

**Table SM1**	*Associations between Actor Power, Perceived Partner Power, and Sexual Compromise Using All Items (Studies 1-3)*

| Predictors | *Enacted Compromise* | | | | | *Willingness to Compromise* | | | | |
| --- | --- | --- | --- | --- | --- | --- | --- | --- | --- | --- |
|  | *b* | 95% CI | *β/r* | *t* | *p* | *b* | 95% CI | *β/r* | *t* | *p* |
| *Study 1* |  |  |  |  |  |  |  |  |  |  |
| Actor Power | .04 | -.18, .26 | .03 | 0.35 | .730 | .12 | -.02, .27 | .11 | 1.70 | .090 |
| Perceived Partner Power | .15 | -.12, .42 | .08 | 1.10 | .272 | **.45** | **.27, .63** | **.31** | **4.97** | **<.001** |
| Actor Power x Perceived Partner Power | .02 | -.21, .25 | .01 | 0.17 | .869 | **.15** | **.00, .31** | **.12** | **2.00** | **.047** |
| Gender | .18 | -.02, .37 | .12 | 1.76 | .080 | .31 | .18, .44 | .29 | 4.74 | <.001 |
| Gender x Actor Power | -.19 | -.41, .03 | -.12 | -1.68 | .095 | -.01 | -.15, .14 | -.01 | -0.11 | .909 |
| Gender x Perceived Partner Power | -.04 | -.31, .23 | -.02 | -0.28 | .781 | -.03 | -.21, .15 | -.02 | -0.34 | .733 |
| Gender x Actor Power x Perceived Partner Power | -.02 | -.24, .21 | -.01 | -0.15 | .881 | -.10 | -.25, .06 | -.08 | -1.26 | .210 |
| *Study 2* |  |  |  |  |  |  |  |  |  |  |
| Actor Power | **-.24** | **-.42, -.06** | **-.16** | **-2.65** | **.009** | -.01 | -.14, .13 | .01 | -0.12 | .908 |
| Perceived Partner Power | -.04 | -.25, .18 | -.02 | -0.36 | .722 | **.28** | **.12, .44** | **.19** | **3.37** | **<.001** |
| Actor Power x Perceived Partner Power | .02 | -.17, .20 | .01 | 0.18 | .854 | .03 | -.12, .17 | .02 | 0.40 | .687 |
| Gender | .19 | .02, .36 | .17 | 2.22 | .028 | .09 | -.05, .22 | .10 | 1.28 | .201 |
| Gender x Actor Power | .03 | -.15, .21 | .02 | 0.35 | .727 | -.02 | -.15, .12 | -.01 | -0.22 | .828 |
| Gender x Perceived Partner Power | .04 | -.18, .25 | .02 | 0.34 | .733 | .07 | -.09, .24 | .05 | 0.90 | .367 |
| Gender x Actor Power x Perceived Partner Power | -.06 | -.24, .13 | -.04 | -0.59 | .556 | .01 | -.13, .16 | .01 | 0.20 | .842 |
| *Study 3* |  |  |  |  |  |  |  |  |  |  |
| Actor Power | **-.21** | **-.39, -.02** | **-.12** | **-2.20** | **.028** | .02 | -.10, .15 | .02 | 0.37 | .713 |
| Perceived Partner Power | .03 | -.18, .25 | .02 | 0.31 | .759 | **.28** | **.14, .42** | **.21** | **3.85** | **<.001** |
| Actor Power x Perceived Partner Power | .05 | -.14, .23 | .02 | 0.48 | .630 | -.10 | -.22, .02 | -.08 | -1.62 | .106 |
| Gender | .16 | -.01, .33 | .10 | 1.90 | .058 | .30 | .19, .42 | .27 | 5.30 | <.001 |
| Gender x Actor Power | .30 | .11, .48 | .18 | 3.13 | .002 | **.12** | **-.00, .24** | **.10** | **1.95** | **.052** |
| Gender x Perceived Partner Power | -.22 | -.43, -.01 | -.12 | -2.02 | .044 | -.10 | -.24, .05 | -.07 | -1.32 | .187 |
| Gender x Actor Power x Perceived Partner Power | -.07 | -.26, .11 | -.04 | -0.79 | .431 | -.12 | -.24, .01 | -.09 | -1.86 | .064 |

*Note*. Significant effects involving power are shown in bold. Gender = -1 women, 1 men. Studies 1 and 3 involved multiple regression analyses of individuals producing *β* as indicator of standardized effect size. Study 2 involved multilevel regression analyses of dyads with *r* effect sizes calculated as an indicator of effect size.

**Correlations Across Variables (Studies 1-3)**

**Table SM2** *Correlations Between Measures (Study 1)*

| Variable | 1 | 2 | 3 | 4 | 5 | 6 |
| --- | --- | --- | --- | --- | --- | --- |
| 1. Actor Power | -- |  |  |  |  |  |
| 2. Perceived Partner Power | .40** | **--** |  |  |  |  |
| 3. Comfort Initiating Sex | .30** | .24** | -- |  |  |  |
| 4. Comfort Refusing Sex | .35** | .15* | .25** | -- |  |  |
| 5. Enacted Compromise | .03 | .16* | .11 | -.02 | -- |  |
| 6. Willingness to Compromise | .14* | .28** | .22** | .18* | .24** | -- |
| 7. Gender (-1 women, 1 men) | -.23** | -.13* | -.02 | -.21** | .09 | .18** |

*Note.* * *p* < .05 ** *p* < .01.

**Table SM3** *Correlations Between Measures (Study 2)*

| Variable | 1 | 2 | 3 | 4 | 5 | 6 | 7 |
| --- | --- | --- | --- | --- | --- | --- | --- |
| 1. Actor Power | -- |  |  |  |  |  |  |
| 2. Perceived Partner Power | .26** | -- |  |  |  |  |  |
| 3. Sexual Assertiveness | .27** | .13* | -- |  |  |  |  |
| 4. Sexual Compliance | -.27** | -.17* | -.36** | -- |  |  |  |
| 5. Enacted Compromise | -.22** | -.08 | .13* | .12* | -- |  |  |
| 6. Willingness to Compromise | .06 | .17* | .14* | -.03 | .09 | -- |  |
| 7. Sexual Communal Strength | .04 | .25** | .24** | -.01 | .18* | .33** | -- |
| 8. Gender (-1 women, 1 men) | -.17** | .12* | .07 | -.11 | .10 | .14* | .27** |

*Note.* * *p* < .05 ** *p* < .01.

**Table SM4** *Correlations Between Measures (Study 3)*

| Variable | 1 | 2 | 3 | 4 | 5 | 6 | 7 | 8 | 9 | 10 | 11 | 12 |
| --- | --- | --- | --- | --- | --- | --- | --- | --- | --- | --- | --- | --- |
| 1. Actor Power | -- |  |  |  |  |  |  |  |  |  |  |  |
| 2. Perceived Partner Power | .49** | -- |  |  |  |  |  |  |  |  |  |  |
| 3. Comfort Initiating Sex | .20** | .11* | -- |  |  |  |  |  |  |  |  |  |
| 4. Comfort Refusing Sex | .21** | .14** | .18** | -- |  |  |  |  |  |  |  |  |
| 5. Sexual Assertiveness | .32** | .17** | .59** | .27** | -- |  |  |  |  |  |  |  |
| 6. Sexual Compliance | -.21* | -.24** | -.27** | -.31** | -.43** | -- |  |  |  |  |  |  |
| 7. Sexual Acquiescence | -.17* | -.16** | -.11* | -.18** | -.17** | .46** | -- |  |  |  |  |  |
| 8. Enacted Compromise | -.09 | 01 | -.04 | -.12* | -.12* | .18** | .18** | -- |  |  |  |  |
| 9. Willingness to Compromise | .14* | .25** | .20** | .16** | .27** | -.15* | -.06 | .09 | -- |  |  |  |
| 10. Sexual Communal Strength | .16* | .29** | .35** | -.10* | .38** | -.17* | -.14* | .04 | .41** | -- |  |  |
| 11. Sexual Understanding | .10 | .15** | .15** | .18** | .31** | -.14** | -.08 | .02 | .15** | .15** | -- |  |
| 12. Sexual Enticement | -.08 | -.12* | .18** | -.23** | .15** | .15** | .28** | .20** | .08 | .21** | .06 | -- |
| 13. Gender (-1 women, 1 men) | -.08 | -.04 | .19** | -.23** | .13* | -.16** | -.08 | .03 | .16** | .37** | -.08 | .24** |

*Note.* * *p* < .05 ** *p* < .01.

**Further Details and Figures of Actor X Perceived** **Partner Power Interactions (Studies 1-3)**

We describe the significant actor power X perceived partner power interactions in the paper by presenting simple effects from decomposing the interactions at -1 *SD* and +1 *SD* levels of actor and perceived partner power (see Preacher et al., 2006; we provide syntax on OSF for calculating and running models to provide the effects and predicted values at low and high levels of actor and partner power). Here we also present figures to accompany the necessarily brief descriptions in the paper to provide additional illustration of our interpretation of the interactions. As we summarize in the paper, there was no evidence that the effects of power hinge on power asymmetries involving actors having low and partners having high perceived power, or vice versa. Instead, actor and perceived partner power consistently had significant distinct effects, actor x perceived partner interactions only emerged in a small proportion of tests (6 of 19 analyses), and the pattern of interactions did not support that sexual inhibition or neglect were greatest in the context of perceived power asymmetries.

**Study 1**

In two of four outcomes in Study 1, significant interactions between actor power and perceived partner power emerged. First, the actor power x perceived partner power interaction was significant (*p* = .050) when predicting *comfort refusing sex*. As shown in Figure SM1, the positive association between actor power and comfort refusing sex was stronger when perceived partner power was also high (+1*SD*: *b* = .60, *t* = 5.48, *p* < .001) compared to when perceived partner power was low (-1*SD*: *b* = .29, *t* = 2.02, *p* = .045). Rather than sexual assertiveness being greater in the context of perceived power asymmetries (high actor power-low perceived partner power), this pattern indicates that low perceived partner power may stifle the sexual assertiveness of high power actors, although differences at high actor power were small.

*Figure SM1. Significant Interaction between Actor Power and Perceived Partner Power on Comfort Refusing Sex (Study 1).*

*Note.* Low and high levels are plotted at 1 SD below and 1 SD above the mean. We also plot the y axis from approximately 1 SD below to 1 SD above the mean. Error bars reflect standard errors.

Second, the actor power x perceived partner power interaction was significant (*p* = .015) when predicting *willingness to compromise*. As shown in Figure SM2, the positive association between perceived partner power and willingness to compromise was stronger when actor power also was high (+1*SD*: *b* = .77, *t* = 4.74, *p* < .001) compared to when actor power was low (-1*SD*: *b* = .31, *t* = 2.36, *p* = .019). Rather than willingness to compromise being greater in the context of perceived power asymmetries (high perceived partner power X low actor power), this pattern indicates that willingness to compromise is greater when both partners enjoy high power.

*Figure SM2. Significant Interaction between Actor Power and Perceived Partner Power on Willingness to Compromise (Study 1).*

*Note.* Low and high levels are plotted at 1 SD below and 1 SD above the mean. We also plot the y axis from approximately 1 SD below to 1 SD above the mean. Error bars reflect standard errors.

**Study 2**

Across the five outcomes examined in Study 2, there were no significant interactions between actor power and perceived partner power. In additional analyses examining partner-reported instead of perceived partner power (see Additional Analyses, Partner-Reported Power in this document), there were significant gender differences in the actor x partner-reported power interaction predicting sexual compliance. For men, a significant actor x partner-reported power interaction (*p* = .040) indicated that men low in actor power reported greater sexual compliance when their women partner also reported they had low but not high power (see further details in Additional Analyses below). This pattern does not support that power asymmetries produce compliance, and instead continues to support the more general theme that low power impedes healthy sexual behavior, including promoting compliance.

**Study 3**

Across the 10 outcomes examined in Study 3, there were 4 interactions between actor power and perceived partner power, two of which emerged for men only.

First, as in Study 1, the actor x perceived partner power interaction was significant when predicting *comfort refusing sex,* but this interaction significantly differed by gender. The significant actor x perceived partner power for men (*b* = .45, 95% CI [.17, .73], *β* = .22, *t* = 3.13, *p* = .002) is shown in Figure SM3. The interaction was similar to that of Study 1. For men, the positive association between actor power and comfort refusing sex was stronger when perceived partner power was high (+1*SD*: *b* = .70, 95% CI [.31, 1.06], *β* = .35, *t* = 3.65, *p* < .001) and was not significant when perceived partner’s power was low (-1*SD*: *b* = -.06, 95% CI [-.45, .33], *β* = -.03, *t* = -0.32, *p* = .752), indicating that low perceived partner power stifles rather than amplifies the sexual assertiveness of high power men. For women, the actor x perceived partner power was not significant (*b* = .04, 95% CI [-.21, .27], *β* = .02, *t* = 0.28, *p* = .777). Instead, for women, the positive association between actor power and comfort refusing sex did not significantly differ when perceived partner power was high (+1*SD*: *b* = .30, 95% CI [.01, .59], *β* = .20, *t* = 2.01, *p* < .001) or low (-1*SD*: *b* = .24, 95% CI [-.08, .55], *β* = .16, *t* = 1.49, *p* = .137).

*Figure SM3. Significant Interaction between Actor Power and Perceived Partner Power on Men’s Comfort Refusing Sex (Study 3).*

*Note.* Low and high levels are plotted at 1 SD below and 1 SD above the mean. We also plot the y axis from approximately 1 SD below to 1 SD above the mean. Error bars reflect standard errors.

Second, the actor x perceived partner power interaction also was significant (*p* = .008) when predicting *sexual assertiveness*, which revealed the same pattern as comfort refusing sex in Study 1 and (for men) Study 3. As shown in Figure SM4, the positive association between actor power and assertiveness was stronger when perceived partner power also was high (+1*SD*: *b* = .33, *t* = 6.50, *p* < .001) versus low (-1*SD*: *b* = .15, *t* = 2.81, *p* = .005).

*Figure SM4. Significant Interaction between Actor Power and Perceived Partner Power on Sexual Assertiveness (Study 3).*

*Note.* Low and high levels are plotted at 1 SD below and 1 SD above the mean. We also plot the y axis from approximately 1 SD below to 1 SD above the mean. Error bars reflect standard errors.

*Figure SM5. Significant Interaction between Actor Power and Perceived Partner Power on Men’s Willingness to Compromise (Study 3).*

*Note.* Low and high levels are plotted at 1 SD below and 1 SD above the mean. We also plot the y axis from approximately 1 SD below to 1 SD above the mean. Error bars reflect standard errors.

Third, the actor and actor x perceived partner power interaction when predicting *willingness to compromise* significantly differed by gender. For men, higher actor power also predicted greater willingness to compromise (*b* = .22, 95% CI [.02, .41], *β* = .17, *t* = 2.19, *p* = .029), but this was accompanied by a marginally significant interaction (*b* = -.16, 95% CI [-.34, .03], *β* = -.12, *t* = -1.68, *p* = .095). As shown in Figure SM5, for men, actor power only was associated with greater willingness to compromise when perceived partner power was low (-1*SD*: *b* = .35, 95% CI [.09, .61], *β* = .27, *t* = 2.70, *p* = .005) but not high (+1*SD*: *b* = .09, 95% CI [-.16, .33], *β* = .07, *t* = .70, *p* = .486, right side of figure). By contrast, for women, neither actor power (*b* = -.08, 95% CI [-.27, .12], *β* = -.06, *t* = -.82, *p* = .417), nor the interaction between actor and perceived partner power (*b* = .16, 95% CI [-.05, .36], *β* = .10, *t* = 1.50, *p* = .133), was significant. Taken with the significant perceived partner power effect that did not differ across gender, the overall results indicate that both women and men were more willing to compromise to high perceived power partners, but high power men also appeared to be motivated to attend to low perceived power partners.

Fourth, the actor x perceived partner power interaction when predicting *sexual understanding* was significant (*p* <.001). As shown in Figure SM6, the positive association between perceived partner power and sexual understanding was significant when actor power also was high (+1*SD*: *b* = .38, *t* = 4.27, *p* < .001) but not when actor power was low (-1*SD*: *b* = .02, *t* = 0.29, *p* = .769). Thus, sexual understanding was greatest when both actor and perceived partner power were high.

*Figure SM6. Significant Interaction between Actor Power and Perceived Partner Power on Willingness to Compromise (Study 3).*

*Note.* Low and high levels are plotted at 1 SD below and 1 SD above the mean. We also plot the y axis from approximately 1 SD below to 1 SD above the mean. Error bars reflect standard errors.

**Summary of Actor x Perceived** **Partner Interactions Across Studies**

Actor power x perceived partner interactions only emerged in 6 of 19 analyses across studies. Moreover, in no cases did the pattern indicate that sexual approach–inhibition or accommodation–neglect emerge from asymmetries involving low actor power and high perceived partner power. With regard to approach–inhibition outcomes, three (of 9 possible) interactions predicting *comfort refusing sex* (Study 1 and Study 3, men only) and *sexual assertiveness* (Study 3 but not Study 2) revealed that higher actor power promotes greater comfort refusing sex and sexual assertiveness, but such approach behavior can be stifled rather than magnified when partners are perceived to be low in power.

With regard to accommodation–neglect outcomes, three (of 10 possible) interactions predicting *willingness to compromise* (Study 1 and Study 3, men only) and *sexual understanding* (in Study 3) revealed that accommodation was greater when both perceived partner power and actor power were high, and in one case men high in actor power also were willing to compromise when partners were perceived to be low in power.

**Summary of Additional Analyses Measures and Results (Studies 1-3)**

In each study, we ran additional analyses controlling for alternative explanations, including femininity and masculinity ideology (assessed in Study 1 only), sexual satisfaction (assessed in all studies), and sexual desire (assessed in Studies 2 and 3). Below we briefly describe the assessment of each construct and summarize the results for each study. Given the large number of additional analyses, rather than produce numerous additional tables of results we describe the results and point the reader to the data files and syntax available at <https://osf.io/e54zr/> if readers are interested in reproducing these additional analyses.

**Femininity and Masculinity Ideology (Study 1)**

One prior paper (Harrington & Maxwell, 2023) has used data in Study 1 to assess the effects of femininity and masculinity ideology on sexual assertiveness. Participants completed a short-version of the Femininity Ideology Scale (Levant et al., 2007). Eight items were averaged (e.g., “A woman should not expect to be sexually satisfied by her partner”, “Women should not view/use pornographic material”; α = .78; *M* = 1.57, *SD* = .75). Participants also completed the 5-item Male Role Norms Inventory Very Brief (McDermott et al., 2019; e.g., “A man should always be the boss”; α = .86; *M* = 2.51, *SD* = 1.39). We reran the current analyses examining the links between power and sexual assertiveness controlling for the main and interaction effects of femininity and masculinity ideology as tested in the prior paper. The links between power and sexual assertiveness were unchanged. See Study 1 Files on the project OSF page (link above) to access the data and syntax to replicate these analyses.

**Sexual Satisfaction (Studies 1-3)**

In Study 1, participants completed a 5-item assessment of general sexual satisfaction from the Global Measure of Sexual Satisfaction (Lawrance & Byers, 1995). Participants rated their sex life on 7-point semantic differential scales: Bad—Good, Unpleasant—Pleasant, Negative—Positive, Unsatisfying—Satisfying, Worthless—Valuable. Items were averaged (α = .95; *M* = 5.91, *SD* = 1.19). Higher actor (*r* = .51, *p* < .001) and perceived partner power (*r* = .27, *p* < .001) were associated with greater sexual satisfaction. The links between power and sexual behavior were unchanged when rerunning the primary analyses controlling for sexual satisfaction with one exception: the link between actor power and comfort initiating sex was no longer significant. See Study 1 Files on the project OSF page (link above) to access the data and syntax to replicate these analyses.

In Study 2, participants completed the Quality of Sex Scale (Shaw & Rogge, 2016), which contains two subscales (12 items each) that assess sexual satisfaction (e.g., “My sex life is fulfilling”, “My partner really pleases me sexually”; 0 = *not at all true*, 5 = *completely true*) and sexual dissatisfaction (e.g., “I do not enjoy sexual activity with my partner”, “I am very disappointed with my sex life with my partner). Although highly correlated, Shaw and Rogge (2016) illustrate they are distinct dimensions and we followed their recommendations to sum items from each subscale to create an assessment of sexual satisfaction (α = .96; *M* = 42.12, *SD* = 13.22) and sexual dissatisfaction (α = .88; *M* = 4.15, *SD* = 2.00), which we analyzed separately. Mimicking the primary analyses with satisfaction and dissatisfaction as outcomes revealed that actors higher in power reported greater satisfaction (*t* = 4.27, *p* < .001, *r* = .26) and lower dissatisfaction (*t* = -2.83, *p* = .005, *r* = .17), and actors who perceived their partner to be higher in power reported greater satisfaction (*t* = 3.58, *p* < .001, *r* = .22) and lower dissatisfaction (*t* = -2.93, *p* = .004, *r* = .18). Nonetheless, rerunning the primary analyses controlling for satisfaction or dissatisfaction did not alter the links between power and sexual behaviors and motivations, with one exception: the unexpected link between actor power and enacted compromise was reduced (*p* = .086) when controlling for satisfaction but not dissatisfaction. See Study 2 Files on the project OSF page (link above) to access the data and syntax to replicate these analyses.

In Study 3, participants completed the same 5-item assessment of general sexual satisfaction from the Global Measure of Sexual Satisfaction (Lawrance & Byers, 1995) used in Study 1 (α = .95; M = 5.82, SD = 1.24). Higher actor (*r* = .25, *p* < .001) and perceived partner power (*r* = .20, *p* < .001) were associated with greater sexual satisfaction. Rerunning the primary analyses controlling for sexual satisfaction did not change any of the associations between actor power, perceived partner power, and sexual outcomes with one exception: the actor x perceived partner power predicting sexual assertiveness was no longer significant. See Study 3 Files on the project OSF page (link above) to access the data and syntax to replicate these analyses.

**Sexual Desire (Studies 2 and 3)**

Sexual desire was assessed in Study 2 and 3 only. We controlled for dyadic sexual desire to assess whether any links between power and sexual behavior were the results of a greater interest or wish to engage in sexual activity with the partner. In Study 2, participants completed the Sexual Desire Inventory (SDI; Spector et al., 1996). The assessment of dyadic sexual desire included 8 items assessing desire to engage in sexual activity with a partner (e.g., “When you are in romantic situations (such as a candlelit dinner, a walk on the beach, etc.), how strong is your sexual desire?”, “When you have sexual thoughts, how strong is your desire to engage in sexual behaviour with a partner?”; 1 = *not strong at all*, 7 = *very strong*). Items were summed (α = .80; *M* = 39.29, *SD* = 7.96). Dyadic sexual desire was not associated with actor or partner power. Thus, controlling for dyadic sexual desire did not alter any of the results. See Study 2 Files on the project OSF page (link above) to access the data and syntax to replicate these analyses.

In Study 3, participants completed 3 items from the SDI (Spector et al., 1996) to assess dyadic sexual desire. The three items represented the top loading items that specifically referred to sexual desire toward a partner in prior factor analyses of the sexual desire inventory (Moyano et al., 2017): “How strong is your desire to engage in sexual activity with a partner?”, “When you have sexual thoughts, how strong is your desire to engage in sexual behavior with a partner?”, and “How important is it for you to fulfill your sexual desire through activity with a partner?” (1 = *not strong at all*, 7 = *very strong*). Items were averaged (α = .88; *M* = 5.64, *SD* = 1.28). Higher actor (*r* = .16, *p* < .001) and perceived partner power (*r* = .20, *p* < .001) were associated with greater dyadic sexual desire. Rerunning the primary analyses controlling for dyadic sexual desire did not change any of the associations between actor power, perceived partner power, and sexual behavior and motivations. See Study 3 Files on the project OSF page (link above) to access the data and syntax to replicate these analyses.

**Partner-Reported Power (Study 2)**

Our primary analyses in Study 2 mimicked the tests in Studies 1 and 3 by testing the effects of actor power and perceived partner power. However, because Study 2 included dyads, we could also model the effects of partners reports of their power (partner-reported power). Perceived partner power and partner-reported power were positively correlated as in prior studies (see Overall et al., 2023). However, prior research indicates that perceptions of partner power are likely to be critical in determining behavioral responses because many effects of partner power on actors’ social behavior should occur via perceptions that the partner holds power (e.g., Anderson & Berdahl, 2002; Anderson & Galinsky, 2006; also see Overall et al., 2023). Accordingly, as pre-registered, we expected the effects of partner power to be stronger when examining perceptions of partner power compared to partner-reported power.

We applied Kenny et al.’s (2006) guidelines and SPSS syntax for distinguishable dyads to run Actor-Partner Interdependence Models to simultaneously calculate the main and interaction effects of actor and partner-reported power accounting for the statistical dependence across dyad members. As in the primary analyses modelling perceived partner power, we regressed each outcome on: actor power, partner-reported power, the interaction between actor power and partner-reported power, and the main and interaction effects of gender. To replicate these analyses, access the data and syntax for Study 2 on the project OSF page (link above).

Table SM5 presents the results for all analyses examining partner-reported power instead or perceived partner power. The results for sexual approach–inhibition were the same: higher actor power was associated with greater sexual assertiveness and lower sexual compliance. However, consistent with expectations that perceived partner power should have stronger and more reliable effects, partner-reported power was not significantly associated with sexual compromise or communal strength. These links have important implications for understanding power. If greater accommodation vs. neglect behavior emerges because high power partners force accommodation, then partner-reported power would be expected to have equal or even greater effects. This was not the case highlighting that accommodation vs. neglect behavior emerges to sustain relationships with partners that actors perceive influence valued outcomes (see Overall et al., 2023).

Finally, one significant gender difference emerged for sexual compliance that was not present in the primary analyses modelling perceived partner power. As pre-registered, to assess these significant gender differences, we adopted a two-intercept approach to simultaneously estimate separate effects of actor power, partner-reported power, and actor power x partner-reported power for women and men while continuing to account for dyadic dependence (see Kenny et al., 2006 and syntax on OSF).

As in the primary analyses, the effect of actor power on sexual compliance was significant for both women (*b* = -.19, 95% CI [-.33, -.06], *t* = 2.80, *p* = .006) and men (*b* = -.22, 95% CI [-.33, -.12], *t* = 4.18, *p* < .001). However, differing from the primary analyses, the effect of partner-reported power on compliance significantly differed across women and men.

For men, greater partner-reported power also was weakly associated with lower compliance (*b* = -.12, 95% CI [-.24, .005], *t* = -1.90, *p* = .059) and the interaction between actor and partner-reported power was significant (*b* = .10, 95% CI [.01, .20], *t* = 2.07, *p* = .040). This interaction between actor and partner-reported power for men is illustrated in Figure SM7. Men lower in actor power reported greater sexual compliance when their partner also felt they had lower power (-1*SD*: *b* = -.32, 95% CI [-.46, -.19], *t* = -4.64, *p* < .001, *r* = .36), but not when their partner reported their power was high (+1*SD*: *b* = -.12, 95% CI [-.27, .03], *t* = 1.60, *p =* .113 *r* = .13). This pattern does not support that power asymmetries produce compliance. This pattern does not support that harmful outcomes occur most when one partner is higher in power, and instead continues to support the replicated findings that low power impedes healthy sexual behavior, including promoting compliance.

For women, neither the effect of partner-reported power (*b* = .07, 95% CI [-.05, .19], *t* = 1.20, *p* = .232) or the interaction was significant (*b* = -.026, 95% CI [-.13, .08], *t* = -0.47, *p* = .637). Thus, women lower in power reported greater sexual compliance when partners were low (-1*SD*: *b* = -.16, 95% CI [-.31, -.02], *t* = -2.25, *p* = .026, *r* = .18), and high in power (-1*SD*: *b* = -.22, 95% CI [-.42, -.02], *t* = -2.19, *p* = .03, *r* = .18).

*Figure SM7. Significant Interaction between Actor Power and Partner-Reported Power on Men’s Sexual Compliance (Study 2).*

*Note.* Low and high levels are plotted at 1 SD below and 1 SD above the mean. We also plot the y axis from approximately 1 SD below to 1 SD above the mean of sexual compliance. Error bars represents standard errors.

*Ancillary analyses:* Guided by a reviewer’s curiosity, we also ran analyses simultaneously modelling the effects of perceived partner power (as examined in the primary analyses reported in the paper) and partner-reported power (as examined in the additional analyses described above). The effects of actor and perceived partner power were the same as reported in the paper, and the effects of partner-reported power were the same as reported in Table SM5 below. For full results, interested readers can replicate the analyses by accessing the data and syntax for Study 2 on the project OSF page (link above).

**Table SM5**	*Associations Between Actor Power, Partner Reported Power, and Sexual Behaviors and Motivations (Study 2)*

| Predictors | *b* | 95% CI | *r* | *t* | *p* |
| --- | --- | --- | --- | --- | --- |
| *Predicting Assertiveness* |  |  |  |  |  |
| Actor Power | **.18** | **.11, .26** | **.28** | **4.93** | **<.001** |
| Partner Reported Power | .01 | -.06, .09 | .02 | 0.33 | .739 |
| Actor Power x Partner Reported Power | .02 | -.06, .09 | .03 | 0.41 | .680 |
| Gender | .08 | .02, .14 | .20 | 2.50 | .013 |
| Gender x Actor Power | **.11** | **.03, .18** | **.18** | **2.77** | **.006** |
| Gender x Partner Reported Power | .02 | -.06, .10 | .03 | 0.44 | .659 |
| Gender x Actor Power x Partner Reported Power | -.03 | -.08, .03 | -.08 | -1.02 | .310 |
| *Predicting Sexual Compliance* |  |  |  |  |  |
| Actor Power | **-.21** | **-.29, -.12** | **-.28** | **-4.84** | **<.001** |
| Partner Reported Power | -.02 | -.11, .06 | -.03 | -0.56 | .576 |
| Actor Power x Partner Reported Power | .04 | -.04, .12 | .03 | 0.95 | .342 |
| Gender | -.12 | -.20, -.05 | .25 | -3.20 | .002 |
| Gender x Actor Power | -.01 | -.10, .07 | -.02 | -0.32 | .750 |
| Gender x Partner Reported Power | **-.09** | **-.18, -.01** | **-.13** | **-2.17** | **.031** |
| Gender x Actor Power x Partner Reported Power | .06 | -.01, .13 | .15 | 1.90 | .059 |
| *Predicting Enacted Compromise* |  |  |  |  |  |
| Actor Power | **-.33** | **-.53, -.12** | **-.19** | **-3.13** | **.002** |
| Partner Reported Power | -.16 | -.36, .05 | -.09 | -1.49 | .138 |
| Actor Power x Partner Reported Power | -.08 | -.26, .11 | -.07 | -0.83 | .407 |
| Gender | .15 | -.05, .35 | .13 | 1.52 | .130 |
| Gender x Actor Power | -.06 | -.27, .14 | -.04 | -0.61 | .541 |
| Gender x Partner Reported Power | .09 | -.12, .30 | .05 | 0.84 | .404 |
| Gender x Actor Power x Partner Reported Power | -.05 | -.22, .13 | -.05 | -0.54 | .591 |
| *Predicting Willingness to Compromise* |  |  |  |  |  |
| Actor Power | .12 | -.03, .27 | .09 | 1.53 | .126 |
| Partner Power | -.02 | -.17, .13 | .02 | -0.26 | .792 |
| Actor Power x Partner Reported Power | .02 | -.12, .15 | .02 | 0.21 | .837 |
| Gender | .20 | .06, .34 | .23 | 2.83 | .005 |
| Gender x Actor Power | -.04 | -.20, .12 | -.03 | -0.52 | .602 |
| Gender x Partner Reported Power | -.01 | -.16, .15 | -.01 | -0.85 | .932 |
| Gender x Actor Power x Partner Reported Power | -.03 | -.16, .09 | -.04 | -0.51 | .608 |
| *Predicting Communal Strength* |  |  |  |  |  |
| Actor Power | .07 | -.03, .17 | .08 | 1.38 | .169 |
| Partner Power | .06 | -.04, .17 | .07 | 1.25 | .211 |
| Actor Power x Partner Reported Power | .03 | -.06, .12 | .05 | 0.62 | .538 |
| Gender | .25 | .15, .35 | .39 | 5.12 | <.001 |
| Gender x Actor Power | .04 | -.06, .15 | .05 | 0.83 | .409 |
| Gender x Partner Reported Power | .03 | -.08, .13 | .03 | 0.47 | .638 |
| Gender x Actor Power x Partner Reported Power | -.07 | -.15, .02 | -.13 | -1.54 | .126 |

*Note*. Significant effects involving power are shown in bold. Gender = -1 women, 1 men.

**References**

Ackerman, R.A., Ledermann, T., & Kenny, D.A. (2020). *Power analysis for the actor-partner interdependence model* [Software]*.* <https://robert-ackerman.shinyapps.io/APIMPowerR>

Anderson, C., & Berdahl, J. L. (2002). The experience of power: Examining the effects of power on approach and Inhibition tendencies. *Journal of Personality & Social Psychology, 83(6),* 1362–1377. <https://doi.org/10.1037//0022-3514.83.6.1362>

Anderson, C., & Galinsky, A. D. (2006). Power, optimism, and risk-taking. *European Journal of Social Psychology, 36(4),* 511–536. <https://doi.org/10.1002/ejsp.324>

Balzarini, R. N., Muise, A., Dobson, K., Kohut, T., Raposo, S., & Campbell, L. (2021). The detriments of unmet sexual ideals and buffering effect of sexual communal strength. *Journal of Personality and Social Psychology*, *120*(6), 1521–1550. <https://doi.org/10.1037/pspi0000323>

Burke, T. J., & Young, V. J. (2012). Sexual transformations and intimate behaviors in romantic relationships. *Journal of Sex Research*, *49*(5), 454-463. <https://doi.org/10.1080/00224499.2011.569977>

Cohen, J. (1988). *Statistical power analysis for the behavioral sciences* (2nd ed.). Hillsdale, NJ: Lawrence Erlbaum.

Faul, F., Erdfelder, E., Buchner, A., & Lang, A.-G. (2009). Statistical power analyses using G*Power 3.1: Tests for correlation and regression analyses. *Behavior Research Methods*, *41*, 1149-1160.

Harrington, A. G., & Maxwell, J. A. (2023). It takes two to tango: Links between traditional beliefs about both men’s and women’s gender roles and comfort initiating sex and comfort refusing sex. *Sex Roles*, *88*(11), 514-528. <https://doi.org/10.1007/s11199-023-01366-w>

Hurlbert, D. F. (1991). The role of assertiveness in female sexuality: A comparative study between sexually assertive and sexually nonassertive women. *Journal of Sex & Marital Therapy*, *17*(3), 183–190. <https://doi.org/10.1080/00926239108404342>

Kim, J. J., Muise, A., Sakaluk, J. K., Rosen, N. O., & Impett, E. A. (2020). When tonight is not the night: Sexual rejection behaviors and satisfaction in romantic relationships. *Personality and Social Psychology Bulletin, 46(10),* 1476-1490. <https://doi.org/10.1177/0146167220907469>

Lawrance K. A., Byers E. S. (1995). Sexual satisfaction in long-term heterosexual relationships: The interpersonal exchange model of sexual satisfaction. *Personal Relationships*, 2(4), 267–285. <https://doi.org/10.1111/j.1475-6811.1995.tb00092.x>

Levant, R. F., Alto, K. M., McKelvey, D. K., Richmond, K. A., & McDermott, R. C. (2017). Variance composition, measurement invariance by gender, and construct validity of the femininity ide­ology scale-short form. *Journal of Counseling Psychology*, *64*(6), 708–723. <https://doi.org/10.1037/cou0000230>

McDermott, R. C., Levant, R. F., Hammer, J. H., Borgogna, N. C., & McKelvey, D. K. (2019). Development and validation of a five-item male role norms inventory using bifactor modeling. *Psychology of Men & Masculinities*, *20*(4), 467–477. <https://doi.org/10.1037/men0000178>

MacNeil, S., & Byers, E. S. (1997). The relationships between sexual problems, communication, and sexual satisfaction. *The Canadian Journal of Human Sexuality, 6(4*), 277–284.

Maxwell, J. A., Muise, A., MacDonald, G., Day, L. C., Rosen, N. O., & Impett, E. A. (2017). How implicit theories of sexuality shape sexual and relationship well-being. *Journal of Personality and Social Psychology*, *112*(2), 238-279. [https://doi.org/10.1037/pspi0000078](https://psycnet.apa.org/doi/10.1037/pspi0000078)

Moyano, N., Vallejo-Medina, P., & Sierra, J. C. (2017). Sexual desire inventory: two or three dimensions?. *The Journal of Sex Research*, *54*(1), 105-116. <https://doi.org/10.1080/00224499.2015.1109581>

Muise, A., Impett, E. A., Kogan, A., & Desmarais, S. (2013). Keeping the spark alive: Being motivated to meet a partner’s sexual needs sustains sexual desire in long-term romantic relationships. *Social Psychological and Personality Science*, *4*(3), 267-273. <https://doi-org/10.1177/1948550612457185>

Overall, N. C., Maner, J. K., Hammond, M. D., Cross, E. J., Chang, V. T., Low, R. S. T., Girme, Y. U., Jayamaha, S. D., Reid, C. J., & Sasaki, E. (2023). Actor and partner power are distinct and have differential effects on social behavior. *Journal of Personality and Social Psychology, 124(2*), 311–343. <https://doi.org/10.1037/pspi0000398>

Preacher, K. J., Curran, P. J., & Bauer, D. J. (2006). Computational tools for probing interaction effects in multiple linear regression, multilevel modeling, and latent curve analysis. *Journal of Educational and Behavioral Statistics, 31*, 437-448. <https://doi.org/10.3102/10769986031004437>

Shaw, A. M., & Rogge, R. D. (2016). Evaluating and Refining the Construct of Sexual Quality With Item Response Theory: Development of the Quality of Sex Inventory. *Archives of Sexual Behavior, 45(2)*, 249–270. <https://doi.org/10.1007/s10508-015-0650-x>

Spector, I. P., Carey, M. P., & Steinberg, L. (1996). The Sexual Desire Inventory: Development, factor structure, and evidence of reliability. *Journal of Sex and Marital Therapy, 22*, 175–190. <https://doi.org/10.1080/00926239608414655>
